# Perinatal antibiotics in a db/db mouse model impact obesity and hyperglycemia in a microbiome-dependent manner

**DOI:** 10.3389/frmbi.2026.1750320

**Published:** 2026-07-27

**Authors:** Noelle Curtis-Joseph, Audra Laubi, Melanie Ortiz-Alvarez de la Campa, Peter Belenky

**Affiliations:** 1 Department of Molecular Biology, Cellular Biology, and Biochemistry, Brown University, Providence, RI, United States; 2 Department of Molecular Microbiology and Immunology, Brown University, Providence, RI, United States

**Keywords:** antibiotics, db/db, diabetes, *Duncaniella muris*, early-life microbiome, microbiome, obesity

## Abstract

Obesity affects over one billion people globally; however, the role of the gut microbiome and host genetics in its manifestation is poorly understood. We demonstrate that genetic obesity in leptin-receptor-deficient db/db mice requires a permissive gut microbiome, which is established during early life. Using perinatally-administered antibiotic cocktail treatment until pups were 8-weeks-old, we demonstrate that microbiome perturbation substantially reduces weight gain and significantly reduces hyperglycemia in homozygous, leptin-receptor-deficient db/db (Hom) mice without altering caloric intake or extraction efficiency. 16S rRNA sequencing revealed that antibiotic treatment depletes *Muribaculaceae* while enriching *Akkermansiaceae* and *Bacteroidaceae*. Differential abundance analysis identified *Duncaniella muris*, a recently characterized *Muribaculaceae* species, as the most depleted taxon in antibiotic-treated mice. Oral gavage of cultured *D. muris* into antibiotic-treated db/db mice restored hyperglycemia to pre-treatment levels without affecting body weight, establishing a direct causal link between this specific microbe and glucose increase. These findings reveal that hyperglycemia is not solely genetic, but depends critically on specific microbiota members in a permissive microbial context.

## 1 Introduction

In 2022, over one billion people, or one in eight individuals globally, were living with obesity, while 43% of adults were classified as overweight (World Health Organization, 2024). Among children and adolescents aged 5–19 years, the prevalence of being overweight and obese increased from roughly 4% in 1975 to nearly 20% in 2022 (World Obesity Federation, 2024). To address the rapid rise in obesity, we need to identify modifiable factors that influence risk. For example, the gut microbiome is emerging as a contributor and target of obesity, given its potential for modulation. The gut microbiome is comprised of a dynamic community of metabolically active commensal bacteria that profoundly influence host physiology through mechanisms such as bacterial energy harvest and metabolic signaling (Cani and Jordan, 2018). Microbial-derived short-chain fatty acids (SCFAs) engage with enteroendocrine cells to regulate satiety and glucose metabolism (Caengprasath et al., 2020; Yadav et al., 2013). This community is shaped by diet, host sex, antibiotic exposure, and host genetics.

Compelling evidence now links the gut microbiome to obesity. Germ-free mice receiving fecal microbiota transplantation (FMT) from obese donors develop significantly greater adiposity than those receiving microbiota from lean donors (Ridaura et al., 2013). Conversely, obese mice receiving FMT from lean donors exhibit reduced adiposity and reduced weight gain (Cao et al., 2025). Compositional shifts in the microbiome are hypothesized to alter energy harvest efficiency and carbohydrate fermentation pathways (Smith et al., 2022). These effects are attributed to microbial metabolites—including SCFAs, secondary bile acids, and endotoxins—that directly influence host metabolic inflammation, energy storage, and insulin sensitivity, as well as alter energy harvest through modulated carbohydrate utilization and fermentation pathways (Song et al., 2023).

The maternal gestation period and early infancy represent critical windows for establishing long-term trajectories of offspring microbiome composition, immune function, and metabolic health (Selma-Royo et al., 2020). The maternally transferred microbiome during delivery and lactation exerts profound influences on early-life immune programming and initial microbiome assembly, with consequences persisting into adolescence and adulthood (Galazzo et al., 2020; Selma-Royo et al., 2024; Jašarević et al., 2021). Maternal antibiotic exposure extending into early, postnatal life represents a potent intervention point for reprogramming the offspring gut microbiome (Lynch et al., 2023; Nabhani and Eberl, 2020). Antibiotic administration during these developmental windows does not merely cause transient microbial shifts; it actively reprograms the developmental trajectory of the microbial ecosystem. Because infections during pregnancy and the peripartum period threaten both maternal and fetal health, antibiotics are frequently prescribed in the United States, including intrapartum penicillin or ampicillin prophylaxis for group B *Streptococcus* colonization. β-lactams and aminoglycosides such as ceftriaxone, ampicillin, and gentamicin are used for urinary tract, soft tissue, and intra-amniotic or postpartum infections, while anti-staphylococcal agents such as dicloxacillin or cephalexin are used for lactational mastitis (Wink et al., 2024; Louis-Jacques et al., 2023; Mitchell et al., 2022). Early-life antibiotic exposure can prevent establishment of key bacterial taxa during formative periods when community structure is most flexible (Thorburn et al., 2015; Agüero et al., 2016). Critically, these absent pioneer species cannot be simply replaced later—the ecological opportunity for their integration is lost as they are excluded from a microbial community that would have supported their colonization (Segura Munoz et al., 2022). This loss of early-life taxa and their encoded functions has demonstrated the capacity to permanently alter offspring metabolic outcomes (Agüero et al., 2016; Nakajima et al., 2017). For instance, perinatal antibiotics consistently reduce *Bacteroides* and delay normal maturation of the gut microbiota, whereas later infant antibiotic courses further promote expansion of opportunistic taxa such as *Enterobacteriaceae* and *Streptococcaceae* and maintain altered abundances of key genera including *Bacteroides*, *Faecalibacterium*, and *Dorea* that distinguish exposed from unexposed infants (Ainonen et al., 2021; Zou et al., 2018; Vänni et al., 2021; Torow et al., 2023). However, pups given antibiotics prior to weaning had more *Escherichia-Shigella* and *Enterococcus* in later life (Niu et al., 2020). These differential community structures, established at different developmental milestones, likely engage with host physiology in unique ways, leading to varied phenotypes.

Most microbiome research on obesity has relied on diet-induced obesity (DIO) models, which investigate the impact of transitioning from high fiber, standard chow to Western diets high in fat and sugar (Turnbaugh et al., 2008; Hildebrandt et al., 2009; Bisanz et al., 2019). While it is invaluable to understand how dietary composition shapes the microbial environment, this approach frames diet as the sole driver of obesity, rarely considering genetic predisposition. Rather than observing a microbiome that drives obesity independently of diet, the field has predominantly examined dietary shaping of the microbial environment alone. To address this gap, we employed the leptin-receptor-deficient db/db mouse model of genetic obesity (Suriano et al., 2021). Homozygous db/db mice harbor mutations in the leptin receptor gene, rendering them insensitive to the satiety hormone leptin (Chen et al., 1996). These mice exhibit hyperphagia, hyperinsulinemia, hyperglycemia, and severe adiposity, without the need for a Western diet, providing a stable, host-encoded driver of metabolic dysfunction independent of dietary manipulation. This model enables investigation of how an obesity-predisposed genotype shapes the microbiome to further promote metabolic disease. Evidence suggests that genetic influence on the microbiome is context-dependent (Goodrich et al., 2014; Rothschild et al., 2018). Twin studies have identified heritable taxa such as members of the *Christensenellaceae* family, which are associated with reduced weight gain when supplemented into germ-free mice (Goodrich et al., 2014). However, dietary and external exposures often outweigh genetic effects in determining baseline community structure (Rothschild et al., 2018). The critical role of host genotype may lie not in dictating a baseline microbiome composition, but in modulating the host’s response to perturbation (Fujisaka et al., 2016). External perturbations—such as antibiotic exposure—may yield differential metabolic outcomes depending on host genetic background, presumably mediated through genotype-specific shifts in gut microbiome composition (Safari et al., 2020).

Antibiotic exposure profoundly reshapes the gut microbiome, with effects that predominantly depend on dosage, timing, and host developmental stage. Not all antibiotic treatment is equal—antibiotics are commonly used at therapeutic dosages to eliminate certain bacterial populations; however, they can also be used to temporarily suppress particular taxa at subtherapeutic levels (Spigaglia, 2024; Anthony et al., 2022). Early agricultural literature demonstrated that subtherapeutic antibiotic administration has long been employed as a growth promoter in livestock by consistently increasing weight gain (Branion and Hill, 1951; McGinnis et al., 1951). This practice unconsciously revealed that antibiotic-induced microbial perturbation can enhance adiposity, an effect subsequently replicated in rodent models (Shelton et al., 2023; Forbes and Park, 1959). In humans, epidemiological studies have associated early-life antibiotic exposure with increased obesity risk in later childhood, suggesting lasting metabolic consequences of developmental microbiome disruption (Trasande et al., 2012). The mechanisms underlying these effects likely involve the same pathways through which the microbiome influences metabolic health: altered SCFA production, changes in energy harvest efficiency, and shifts in microbial community that affect host glucose homeostasis and adiposity (Corbin et al., 2023; Ota et al., 2022; Shelton et al., 2023). Antibiotic-induced dysbiosis is linked to insulin resistance, and depletion of butyrate-producing genera such as *Faecalibacterium* and *Roseburia* is associated with impaired insulin homeostasis (Cui et al., 2022; Aggarwal et al., 2022). Conversely, a large human cohort study indicated that *Coprococcus* was significantly associated with higher insulin sensitivity and lower odds of dysglycemia (Cui et al., 2022). Furthermore, epidemiological data have revealed a dose-dependent relationship between increased antibiotic exposure and the risk of developing type 2 diabetes (Park et al., 2021; Nuotio et al., 2022). Paradoxically, antibiotic-induced microbial depletion can improve glucose tolerance in some models by reducing gram-positive bacteria that contribute to metabolic dysfunction and insulin resistance (Aggarwal et al., 2022). However, antibiotic-induced loss of gut microbes can also alter short-chain fatty acid and bile acid metabolism, with context-dependent effects on peripheral insulin sensitivity and glucose homeostasis (Aggarwal et al., 2022).

Perinatal antibiotic exposure—administered during the critical windows of maternal gestation, delivery, and early nursing—may exert profound effects by preventing the establishment of foundational microbial communities during the periods when the microbiome is most malleable. However, the interaction between host genetics and antibiotic-induced microbiome perturbation in determining metabolic outcomes remains largely underexplored, especially in models of genetic obesity where obesity is intrinsic rather than diet-driven.

We hypothesized that in genetically obese mice, obesity may be determined by a combination of genotype as well as microbiome composition. Specifically, obesity may be entirely genetically encoded but may require a specific microbiome state to manifest. We used a broad-spectrum perinatal antibiotic cocktail to suppress the microbiome, consisting of ciprofloxacin (100 mg/L; targeting gram-negative bacteria), vancomycin (300 mg/L; targeting gram-positive bacteria), and amoxicillin (166 mg/L; targeting both gram-positive and some gram-negative bacteria). We defined perinatal antibiotic exposure as antibiotic treatment of dams administered via drinking water before conception, during pregnancy, throughout lactation, and continuing in offspring post-weaning until 8-weeks-of-age. By combining the db/db genetic model with this perinatal antibiotic perturbation, we aimed to identify specific bacterial taxa and communities that contribute or are permissive to obesity pathogenesis. We predicted that this intervention would reveal compositional shifts associated with reduced weight gain in db/db mice while simultaneously identifying pro-obesogenic taxa through 16S rRNA gene sequencing.

## 2 Materials and methods

### 2.1 Mouse husbandry

All mice were housed at Brown University’s Animal Care Facilities, with use approved under IACUC protocol number 23-05-0013. Heterozygous Lepr^db/+^ (Het) mice, strain #000697, females and males, were purchased from Jackson Laboratory at 4-weeks-old. Following a two-week acclimatization to Brown’s facility, mice were randomly placed in breeding cages consisting of one male and one female. Breeding generated the following genotypes: b6/b6, db/b6, and db/db. Cage assignments for perinatal antibiotic exposure were randomized. Genotypes were determined by PCR for the LepR gene by Transnetyx at time of weaning. The perinatal antibiotic regimen included: 100 mg ciprofloxacin, 300 mg vancomycin, and 166 mg amoxicillin per liter of filtered, distilled water, provided in feeding bottles for ad libitum access. Pups remained with both parents until weaning (three to four weeks; 21–27 days), at which point they were grouped into new cages based on sex, age, and treatment, with four to six mice per cage. All animals were fed standard chow (Laboratory Rodent Diet 5001, LabDiet, St. Louis, MO, USA) in their cages, also ad libitum. Less than 3% morbidity occurred among antibiotic-exposed dams and sires, though it was sporadic and not conclusively linked to treatment. Post-weaning, mice from antibiotic-assigned cages continued receiving the same antibiotic solution until 8-weeks-old, after which they were transitioned to untreated water. Mice not given antibiotics remained on normal water until sacrifice.

### 2.2 Sample collection

Fecal samples were collected weekly from weaning (three to four weeks) until 14 weeks of age, with weights recorded. Starting at 6-weeks-old, weekly blood glucose was measured using a Contour One glucose monitor. At 15 weeks, mice were euthanized using isoflurane followed by cervical dislocation. Blood was collected via cardiac puncture, and plasma was isolated by centrifuging blood at 2,000 × g at 2-4 °C, transferred to Eppendorf tubes, and frozen at -80 °C.

### 2.3 Microbial sample preparation and analysis

Fecal pellets stored at -80 °C were extracted using the ZymoBIOMICS DNA Miniprep Kit (#D4300) and quantified via Qubit 4.0 Fluorometer. Amplicons targeting the 16S V4 region were prepared using Earth Microbiome Project barcoded primers 515F and 806R. PCR was carried out using Phusion High-Fidelity DNA Polymerase (NEB), with cycling conditions as follows: 98 °C for 3 minutes; 35 cycles of 98 °C for 45 seconds, 50 °C for 1 minute, 72 °C for 1 minute 30 seconds; final extension at 72 °C for 10 minutes; hold at 4 °C. Libraries were pooled and cleaned using the NucleoSpin Gel and PCR Clean-up Kit (Macherey-Nagel, Düren, Germany, Item #:740609.50). Sequencing of the pooled libraries was conducted at the Rhode Island Genomics and Sequencing Center at the University of Rhode Island (Kingston, RI, USA). In total we sequenced 246 samples with an average of 31,190 reads per sample.

### 2.4 Bioinformatic analysis

Raw sequencing reads were demultiplexed, quality filtered, trimmed, and denoised using DADA2 via QIIME2 (v2024.5). Trimming removed 10 bases from the 5’ ends of both forward and reverse reads. Samples with fewer than 1,000 features were excluded. Taxonomic classification of ASVs (amplicon sequence variants) was done using the SILVA reference database (silva-138-99-nb-classifier) within QIIME2. Relative abundance and alpha diversity metrics were generated in R, while differential abundance was analyzed with MaAsLin2 (v1.14.1). Beta diversity and PERMANOVA were analyzed in R (v4.3.0) using the phyloseq (v1.44.0) and vegan (v2.6.6.1) packages, respectively. Two-way ANOVA for Shannon diversity was conducted using GraphPad Prism (v10.5.0). ASV data was also retrieved using phyloseq, and DESeq2 (v1.40.2) was used for differential abundance analysis.

### 2.5 Co-abundance network analysis

Co-abundance networks were constructed in R (v4.3.0) using phyloseq (v1.44.0), glasso (v1.11), igraph (v2.0.3), and ggraph (v2.2.2). Taxa were filtered to retain those present in at least 10% of samples with a mean relative abundance of at least 0.0001. The filtered count table was transformed using a centered log-ratio (CLR) transformation. Conditional-dependence networks were inferred by graphical lasso from the covariance matrix of CLR-transformed abundances, with lambda set to 0.10, and taxon pairs were connected by an edge when the strength of their partial correlation was 0.05 or greater. Networks were represented as undirected graphs, with edges indicating conditional associations between taxa, node size scaled by degree, and edge type denoting positive or negative partial correlations.

### 2.6 Bomb calorimetry

Fecal pellets were collected, weighed and stored at -80 °C until analysis. Samples were lyophilized overnight and reweighed to determine dry mass. Lyophilized pellets were then analyzed by bomb calorimetry using a 6725 Semi-Micro Calorimeter (Parr Instrument Company). Since pellet mass varied between samples, gross energy content was normalized to dry sample mass. Analyses were performed with 5 mice per genotype per sex.

### 2.7 Supplementation and gavage experiments

*Duncaniella muris* was sourced from DSMZ (DSM 103720) and reconstituted under anaerobic conditions. Freeze-dried cells were revived in 1 mL of anaerobic MGAM, expanded in 10 mL MGAM for 48 hours at 37 °C, and used to inoculate MGAM agar plates. A colony was transferred to 10 mL fresh MGAM broth and incubated for another 48 hours at 37 °C until reaching OD600 = 1. The culture was then centrifuged at 4,000 × g at 4 °C for 10 minutes. The supernatant was discarded anaerobically, and the pellet was resuspended in anaerobic PBS, re-centrifuged and reconstituted in PBS, and adjusted to 10^8^ CFU per mL. Approximately 100 µL aliquots were loaded into syringes fitted with metal gavage needles and transported in sealed anaerobic flasks to Brown’s animal facility. Five female and four male homozygous db/db mice, raised under the same antibiotic regimen described above, had antibiotics terminated at 8 weeks. *D. muris* was gavaged at 8 weeks + 2 days, with baseline fecal samples collected beforehand, and again at 8 weeks + 6 days using a fresh 10^7^ CFU dose.

### 2.8 Universal bacterial 16S and *D. muris* qPCR

DNA was extracted from fecal pellets following the protocol outlined in Section 2.3 of the methods. Universal bacterial 16S gene qPCR was performed using iTaq Universal SYBR Green Supermix (Bio-Rad), with forward primer 5′-ACTCCTACGGGAGGCAGCAGT-3’, and reverse primer, 5’-ATTACCGCGGCTGCTGGC-3’, with 5 mice per genotype. Cycling conditions were 95 °C for 3 minutes, 40 cycles of 95 °C for 15 seconds, and 63 °C for 60 seconds. Standard curves were generated using a 10-fold serial dilution series of template DNA ranging from 10 to 0.001 ng/µL. For D. muris qPCR, we used the forward primer 5’- CGGCAACGGTGAAACTCAAA -3’, and reverse primer 5’- TTTGAGCCCGGGTAAGGTTC-3’, with 4 mice pre-*D. muris* and 8 mice post-*D. muris*. Standard curves were generated using a 10-fold serial dilution series of template DNA ranging from 5 to 0.0005 ng/µL.

### 2.9 Availability of data and materials

FASTQ datasets are publicly available on the NCBI under BioProject ID PRJNA1366719.

## 3 Results

### 3.1 Experimental schematic

**Figure 1:**
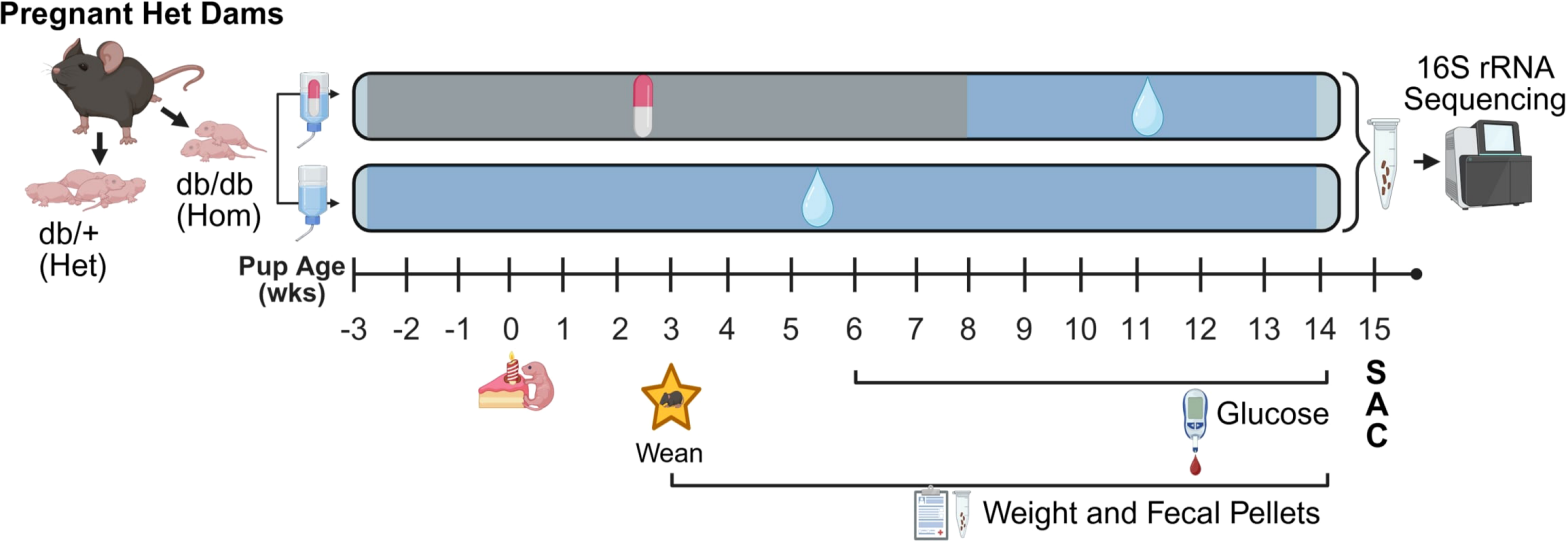
Experimental schematic. Experimental outline of the antibiotic-treatment, and tracking of weight, glucose, and pellet collection. Pink and white pill indicates perinatal antibiotic treatment. Cake indicates pup birth at week 0. Star indicates wean at week 3. Sac = sacrifice. Hom indicates homozygous (db/db) mice. Het indicates heterozygous (db/+) mice.

To understand the impact of genotype and ABX treatment on the gut microbiome of an obesogenic phenotype, we used an early-life maternal manipulation approach to alter the microbiome of Heterozygous, Het (db/+), and Homozygous, Hom (db/db), pups prior to birth up until 8-weeks-old (**Figure 1**). Het females and males were bred in-house to generate a mixture of Het, Hom, and WT mice. To establish distinct microbiomes in the offspring, mating pairs were randomly assigned to either antibiotic cocktail (ABX) in drinking water, or untreated drinking water. The ABX cocktail consisted of ciprofloxacin (100 mg/L), vancomycin (300 mg/L), and amoxicillin (166 mg/L), and was maintained in both mating pairs and their pups after weaning, and until they reached 8-weeks-old, at which point they were switched to untreated water. This early intervention allowed us to establish a different microbial community in the offspring unaffected by microbes transferred from the dam, compared to other studies which have utilized post-weaning antibiotic treatment (Lynch et al., 2023). Other work has shown that prenatal and intrapartum ABX in dams changes the gut microbial composition and intestinal environment in pups (Chen et al., 2021). Maternal ABX treatment disrupts maternal microbiome composition, leading to changes in the SCFAs produced and transferred to the fetus. This disruption produces a long-term, sustained perturbation (Chen et al., 2021; Qin et al., 2025; Madany et al., 2022). To determine the bacterial load in the microbiome of each group of mice, we performed qPCR for the 16S gene and found that there was no significant difference in the copies of bacteria per milligram of fecal material in the microbiome (**Supplementary Figure 1**). This bacterial load recovery may reflect a depletion of antibiotic-sensitive taxa in ABX-treated dams before transmission to pups, allowing surviving taxa more tolerant of antibiotics to colonize and expand in pups by 5-weeks-old, preserving total bacterial load despite altered composition and later continued antibiotic exposure.

To assess the microbiome over time, we sequenced bacterial DNA from fecal pellets collected at four timepoints (ages 5-, 8-, 10-, and 13 weeks). We weighed mice from 3- or 4-weeks-old to 14-weeks-old, and measured glucose levels weekly from 6 to 14 weeks (when the mice were large enough for weekly sampling without distress) (**Figure 1**). This experimental design enabled us to observe both genotype-driven and microbiome-mediated contributions to host weight and glucose, via early-life microbial shifting.

### 3.2 Antibiotics perturb obesity and hyperglycemia in db/db but not Het littermates

**Figure 2:**
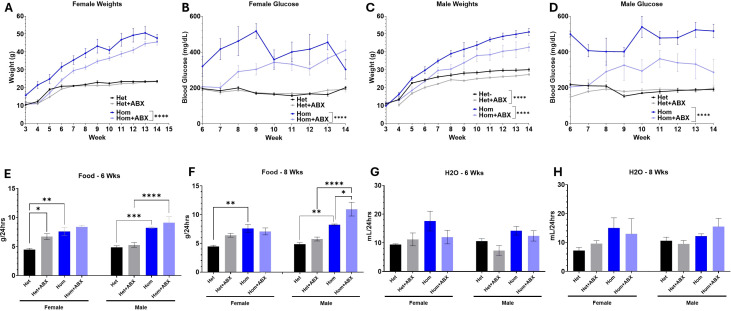
Antibiotics perturb obesity and hyperglycemia in db/db but not Het littermates. (**A)** Female weights. Significance reflects each animal’s mean value across the full longitudinal window shown and was calculated using a mixed-effects Two-way ANOVA. *n* = 4–16 per timepoint per genotype. **(B)** Female glucose. Significance reflects each animal’s mean value across the full longitudinal window shown and was calculated using a mixed-effects Two-way ANOVA. *n* = 4–16 per timepoint per genotype. **(C)** Male weight. Significance reflects each animal’s mean value across the full longitudinal window shown and was calculated using a mixed-effects Two-way ANOVA. *n* = 4–16 per timepoint per genotype. **(D)** Male glucose. Significance reflects each animal’s mean value across the full longitudinal window shown and was calculated using a mixed-effects Two-way ANOVA. *n* = 4–16 per timepoint per genotype. **(E)** Food consumption monitored over 24 hours at 6-weeks-old. Significance based on each animal’s mean value across the full longitudinal window shown. Significance calculated using Two-way ANOVA across genotype and antibiotic treatment with Tukey’s multiple-comparisons test. *n* = 5 per group. **(F)** Food consumption monitored over 24 hours at 8-weeks-old. Significance calculated using Two-way ANOVA across genotype and antibiotic treatment with Tukey’s multiple-comparisons test. *n* = 5 per group. **(G)** Water consumption monitored over 24 hours at 6-weeks-old. Significance calculated using Two-way ANOVA across genotype and antibiotic treatment with Tukey’s multiple-comparisons test. *n* = 5 per group. **(H)** Water consumption monitored over 24 hours at 8-weeks-old. Significance calculated using Two-way ANOVA across genotype and antibiotic treatment with Tukey’s multiple-comparisons test. *n* = 5 per group. Significance values as follows: ns p ≥ 0.05, *p = 0.01 to 0.05, **p = 0.001 to 0.01, ***p = 0.0001 to 0.001, ****p < 0.0001. Hom indicates homozygous (db/db) mice. Het indicates heterozygous (db/+) mice.

Consistent with prior literature, Hom mice exhibited significantly higher body weights than Het controls (Burke et al., 2017). However, Hom+ABX mice did not gain as much weight compared to untreated Hom mice. In both male and female cohorts, ABX treatment led to a significant reduction in weight gain in Hom mice and this impact continued even after the cessation of ABX (week 8) (**Figures 2A, C**). Female Het mice remained largely unaffected. Unexpectedly, we observed that male Het+ABX mice had a significant reduction in longitudinal weight gain compared to untreated Het, suggesting that the impact of antibiotic treatment on weight is sex-specific in Het mice (**Figure 2C**). Wildtype (WT) mice showed a similar weight and glucose pattern as Het mice in both males and females, where they did not have a significant difference in treated and untreated mice (**Supplementary Figures 2A–D**). However, we excluded WT mice from further analysis due to low numbers produced during breeding (**Supplementary Figures 2A–D**). All Hom mice displayed hyperglycemia in untreated conditions, but ABX treatment significantly reduced glucose concentrations (**Figures 2B, D**). Week 9 in female untreated Hom mice showed a sharp drop in glucose (**Figure 2B**). However, these mice are hyperphagic, so the drop in glucose level may represent an advanced stage of metabolic syndrome and dysregulated feeding. As expected, Het mice did not have elevated glucose levels and ABX did not lead to a significant reduction from baseline (**Figures 2B, D**). In summary, we found that ABX treatment slows weight gain in Hom mice, as well as—but to a smaller extent—in male Het mice. On the other hand, glucose was decreased by ABX, but only in the Hom mice. These findings indicate that the anti-obesogenic and anti-hyperglycemic impacts of microbiome disruption depend on both host genotype and sex.

Next, to determine if the reduced weight gain observed in ABX-treated mice was attributed to the mice eating or drinking less under ABX conditions, we monitored food and water consumption of all mouse groups over a 24-hour period (**Figures 2E–H**). Due to being hyperglycemic and hyperphagic, it was expected that Hom mice would generally eat more food and drink more water than Het mice (Cohen et al., 2001). Prior studies showed that whereas Het mice drank just under 10 mL of plain water, and ate 5 g of food per day, Hom mice drank roughly 50 mL and ate 10 g per day (Li et al., 2011). We observed that overall, Hom mice ate and drank more food and water than Het mice at 6- and 8-weeks-old—consistent with the prior literature concerning the hyperphagic and diabetic phenotype of Hom mice (**Figures 2E–H**; Zhu et al., 2022; Sharma et al., 2003). At 6-weeks-old, Het+ABX females exhibited a small, but significant increase in food consumption (**Figure 2E**). Male Het+ABX displayed a significant reduction in weight with ABX-treatment yet ate more and drank less than untreated Hets (**Figures 2C, E–H**). On the other hand, Hom+ABX males consumed significantly more food at 8 weeks compared to all other groups, including untreated Hom mice (**Figures 2E, F**). Despite this elevated intake, Hom+ABX mice gained less weight than untreated Hom mice, suggesting that reduced food intake does not account for antibiotic-induced weight change. We further observed that significantly increased food intake coincided with increased water intake (**Figures 2G, H**). Water intake was variable and trended higher in Hom mice, consistent with their diabetic state. Additionally, at 6-weeks-old, ABX did not significantly change water consumption, indicating that ABX did not deter water intake (**Figure 2G**). To determine if the mice were differentially extracting caloric nutrients from their food, we performed bomb calorimetry on their fecal pellets to calculate calories per milligram of fecal pellet. Our data revealed no differences in energy extraction across groups, indicating that differential caloric absorption likely does not account for the observed phenotypes (**Supplementary Figure 3**).

Taken together, our data demonstrate that ABX selectively reduces weight gain and hyperglycemia in mice based on genotype and sex. The repeated effect of ABX in Hom mice across sexes, combined with the similar food consumption between treated and untreated Hom mice, suggests that microbial perturbation does not impact intake and calorie absorption. Thus, microbiome perturbation is responsible for some of the weight-gain observed in db/db mice. This suggests that the gut microbial community is a critical effector of genotype-driven obesity and hyperglycemia.

### 3.3 Genotype has a significant impact on microbiome composition

**Figure 3:**
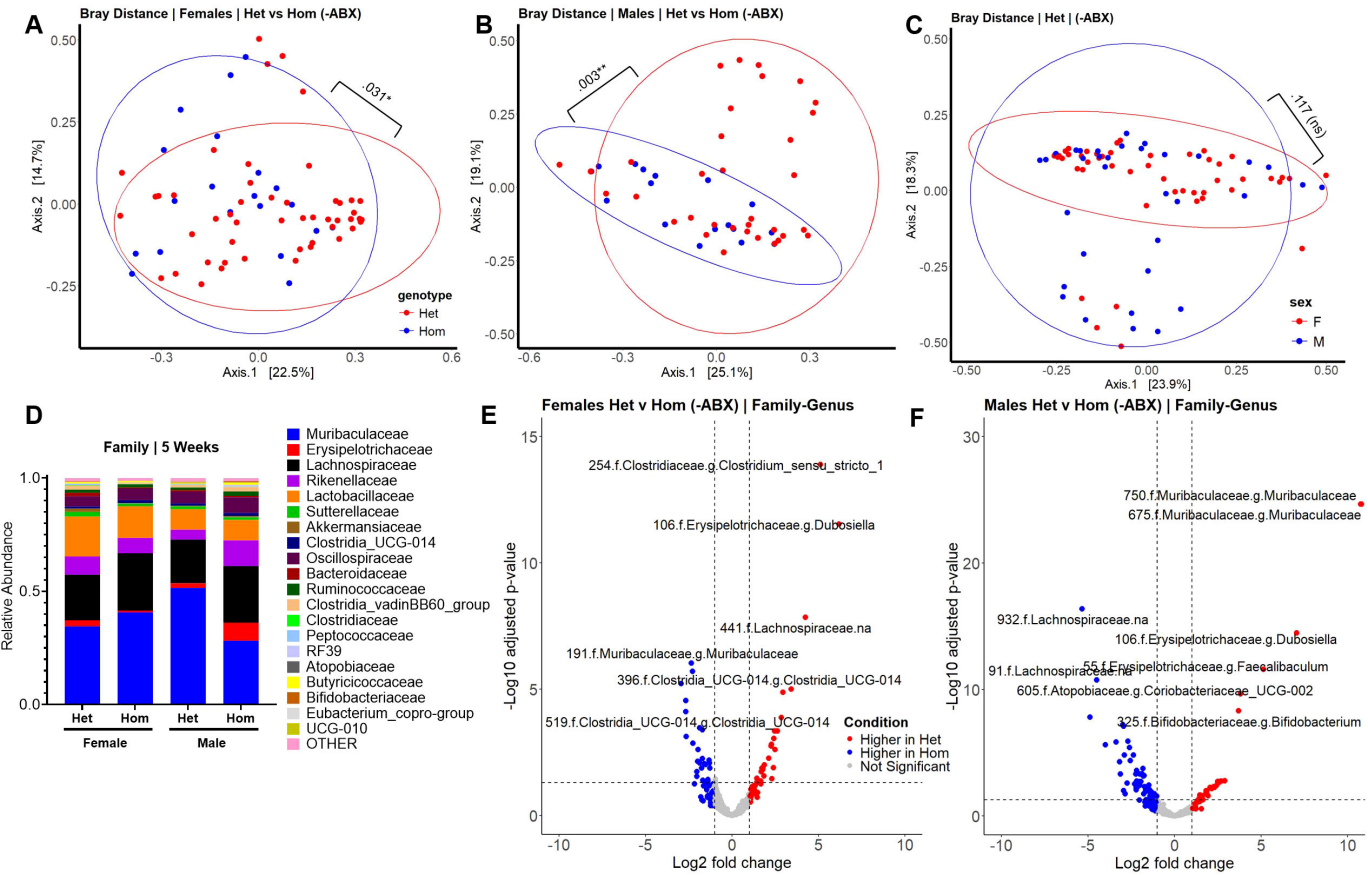
Genotype has a significant impact on microbiome composition. **(A)** Bray-Curtis dissimilarity of untreated Het versus Hom females. *n* = 20-49. **(B)** Bray-Curtis dissimilarity of untreated Het versus Hom males. *n* = 17-34. **(C)** Bray-Curtis dissimilarity of untreated Het females versus males. *n* = 34-49. [**(A–C)** significance calculated using PERMANOVA]. **(D)** Relative abundance at family level of untreated Het and Hom females and males after weaning at 5-weeks-old. *n* = 6-16. **(E)** DESeq2 of untreated Hom versus Het females across all timepoints using individual ASVs. *n* = 20-49. **(F)** DESeq2 of untreated Hom versus Het males across all timepoints using individual ASVs. *n* = 17-34. Significance values as follows: ns p ≥ 0.05, *p = 0.01 to 0.05, **p = 0.001 to 0.01, ***p = 0.0001 to 0.001, ****p < 0.0001.

To determine whether genotype influences microbiome composition in baseline conditions, we performed Bray–Curtis dissimilarity analysis on fecal samples from non-ABX mice (**Figures 3A, B**). We hypothesized that due to the phenotypic differences displayed by the two genotypes, they would have a different microbial structure when compared to each other (**Figures 2A–D**, **3A, B**). We observed that overall, Het and Hom mice have significantly different beta diversity, but not alpha diversity, suggesting that genotype shapes microbial community structure without broadly altering diversity (**Supplementary Figures 4**, **5**). When separated by sex, male and female Hets and Homs displayed a statistically significant divergence in microbiome composition (**Figures 3A, B**). Bray-Curtis further showed us that the significant decrease in weight observed in male Het+ABX, but not female Hets, in **Figure 2C** was not due to a difference in the baseline microbial composition between male and female Hets (**Figure 3C**). Relative abundance profiling at 5-weeks-old revealed *Muribaculaceae* as the dominant family across all groups (**Figure 3D**). Interestingly, female Het mice exhibited lower relative *Muribaculaceae* abundance compared to their Hom counterparts, while in males, the trend was reversed (**Figures 3D–F**). A pronounced taxonomic shift was observed in *Erysipelotrichaceae*, which was enriched in male Hom mice relative to both male Het and all female groups (**Figure 3D**). Consistent with prior studies showing sex-specific microbiome responses, *Lactobacillaceae* were found at higher abundance in females irrespective of genotype (**Figure 3D**; Zhang et al., 2014; Cox et al., 2019).

To better resolve these shifts, differential abundance analysis using DESeq2 identified several genotype-associated taxa (**Figures 3E, F**). Female Het mice exhibited elevated levels of ASVs 254.*Clostridiaceae.clostridium_sensu_stricto1* (*Clostridium celatum*, 99.1% identity), 106.*Erysipelotrichaceae*.g.*Dubosiella* (*Dubosiella newyorkensis*, 100% identity), and 441.*Lachnospiraceae* (*Coprococcus phoceensis*, 97% identity) compared to Hom females (**Figure 3E**; **Supplementary Table 1**). Conversely, female Hom mice were enriched for multiple ASVs within *Muribaculaceae* and *Clostridia_UCG-014*, suggesting a high prevalence of complex glycan-degrading taxa (**Figure 3E**; Sebastià et al., 2024; Smith et al., 2021). Interestingly, *Clostridia_UCG-014* has recently been implicated as a potential gut microbial biomarker for obesity-related early-onset type 2 diabetes mellitus, in humans (Huang et al., 2025). Notably, male Het mice displayed increased abundance of two distinct *Muribaculaceae* ASVs (675 and 750), with high identity to *Clostridium cuniculi*, while male Homs were enriched for 932 and 91.*Lachnospiraceae* (both most similar to *Muricomes intestini*), and showed elevated levels of *Lachnospiraceae NK4A136 group* (**Figure 3F**; **Supplementary Table 1**). Thus, our findings indicate that host genotype determines overall microbiome structure, while exerting subtle, sex-dependent effects on microbial taxa.

### 3.4 Antibiotics have a greater effect on microbiome dissimilarity than genotype

**Figure 4:**
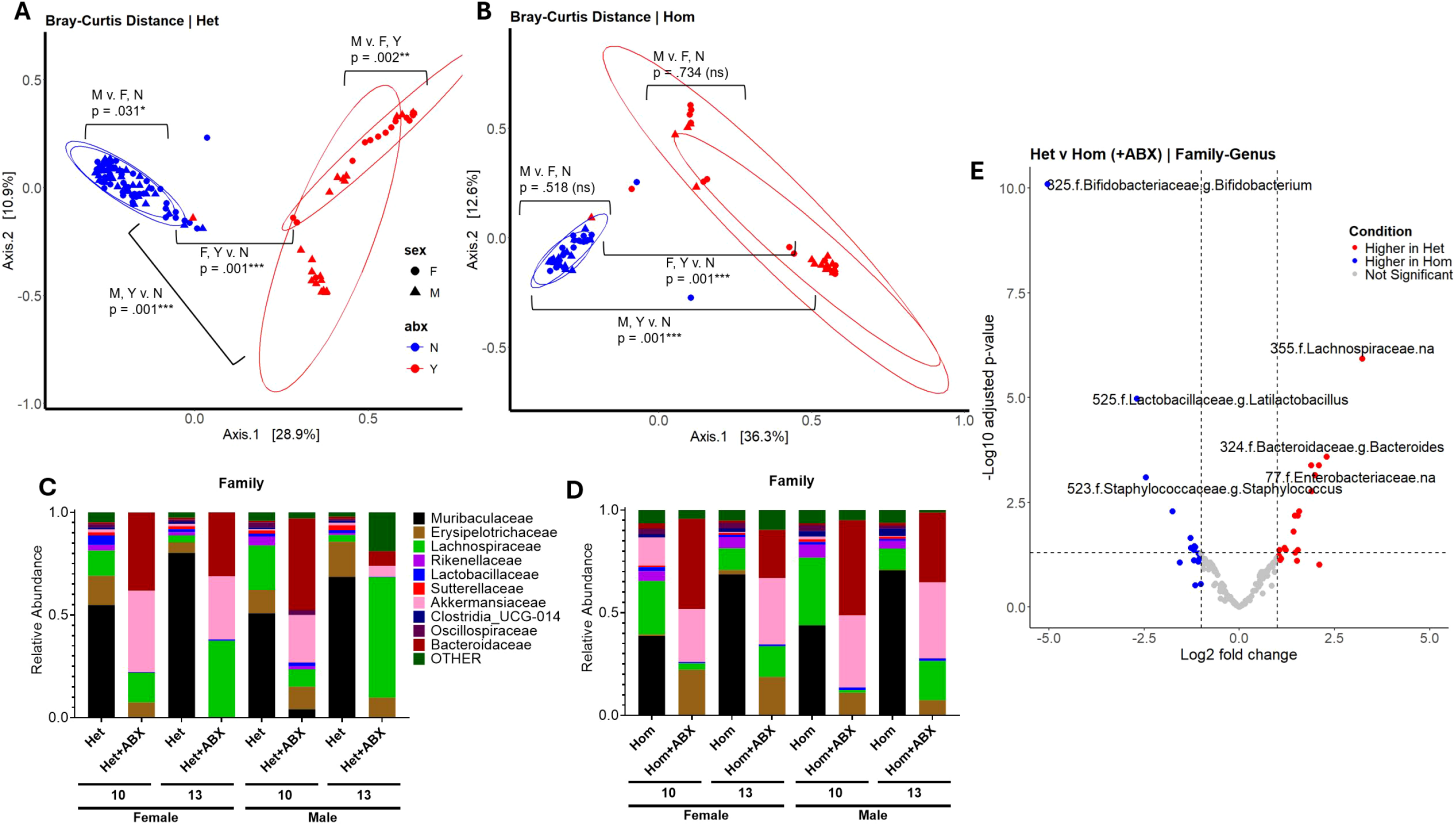
Antibiotics have a greater effect on microbiome dissimilarity than genotype. **(A)** Bray-Curtis dissimilarity of Het mice comparing treated and untreated females and males. *n* = 20-49. **(B)** Bray-Curtis dissimilarity of Hom mice comparing treated and untreated females and males. *n* = 15-20. [**(A, B)**, significance calculated using PERMANOVA]. **(C)** Relative abundance at family level of treated and untreated female and male Het mice during antibiotic recovery at 10- and 13-weeks-old. *n* = 4-11. **(D)** Relative abundance at family level of treated and untreated female and male Hom mice during antibiotic recovery at 10- and 13-weeks old. *n* = 4-11. **(E)** DESeq2 of female and male Het versus Hom treated mice across all timepoints. *n* = 15-20. Significance values as follows: ns p ≥ 0.05, *p = 0.01 to 0.05, **p = 0.001 to 0.01, ***p = 0.0001 to 0.001, ****p < 0.0001.

To determine whether gut microbiome composition was influenced by ABX, we performed Bray-Curtis analysis on Het+ABX and Hom+ABX mice (weeks 5, 8, 10, and 13 combined) (**Figures 4A, B**). Microbiome profiles clustered predominantly by ABX treatment, regardless of sex or genotype. PERMANOVA statistics found a small significant difference between male and female Hets without treatment, with ABX leading to an increased significant difference between males and females (**Figures 4A, B**). When Hom mice were given ABX, we could not detect a significant difference between males and females. This indicates that there may be a genotype-specific, sex-based response of the gut microbiome to ABX. To examine how communities recovered post-ABX, we assessed relative abundance at the family level at 10- and 13-weeks-old.

We observed that after perinatal ABX, at 8-weeks-old, our mice had a microbiome predominantly composed of *Akkermansiaceae* and *Bacteroidaceae* in both males and females of both genotypes (MaAsLin2; Male Het *Bacteroidaceae* coef = 5.2, p < 0.0001; Male Het *Akkermansiaceae* coef = 4.8, p = 0.0011; Male Hom *Bacteroidaceae* coef = 6.3, p < 0.0001; Male Hom *Akkermansiaceae* coef = 4.8, p = 0.0042; Female Het *Bacteroidaceae* coef = 0.5, p = 0.6851; Female Het *Akkermansiaceae* coef = 4.1, p = 0.0286; Female Hom *Bacteroidaceae* coef = 7.5, p < 0.0001; Female Hom *Akkermansiaceae* coef = 3.6, p = 0.0818) (**Supplementary Figures 6A, B**). However, by 10-weeks-old, *Lachnospiraceae* expanded more prominently in Het+ABX mice compared to Hom+ABX, while *Akkermansiaceae* levels remained elevated across all ABX-treated groups, even after treatment cessation (**Figures 4C, D**). Notably, *Erysipelotrichaceae* showed genotype-specific trends where Hom+ABX mice had greater relative abundance than Het+ABX after ABX cessation (**Figures 4C, D**). However, in untreated conditions, Hom mice had lower levels of *Erysipelotrichaceae* compared to Het mice (**Figures 4C, D**). Concurrently, ABX-treated mice displayed decreased *Muribaculaceae* and increased *Bacteroidaceae* abundance during recovery in both genotypes, when compared to their untreated counterparts (**Figures 4C, D**). DESeq2 analysis further confirmed that, under antibiotic conditions, Het+ABX had significantly higher levels of ASVs 355.*Lachnospiraceae*, 324.*Bacteroides*, and 77.*Enterobacteriaceae*, whereas Hom+ABX mice had elevated 325.*Bifidobacterium*, 525.*Latilactobacillus*, and 523.*Staphylococcus* (**Figure 4E**). Thus, these results suggest that antibiotic exposure is the primary driver of microbiome restructuring, while host genotype modulates taxonomic composition during the recovery phase.

### 3.5 Duncaniella muris significantly increases blood glucose to pre-ABX treatment levels in Hom db/db mice

**Figure 5:**
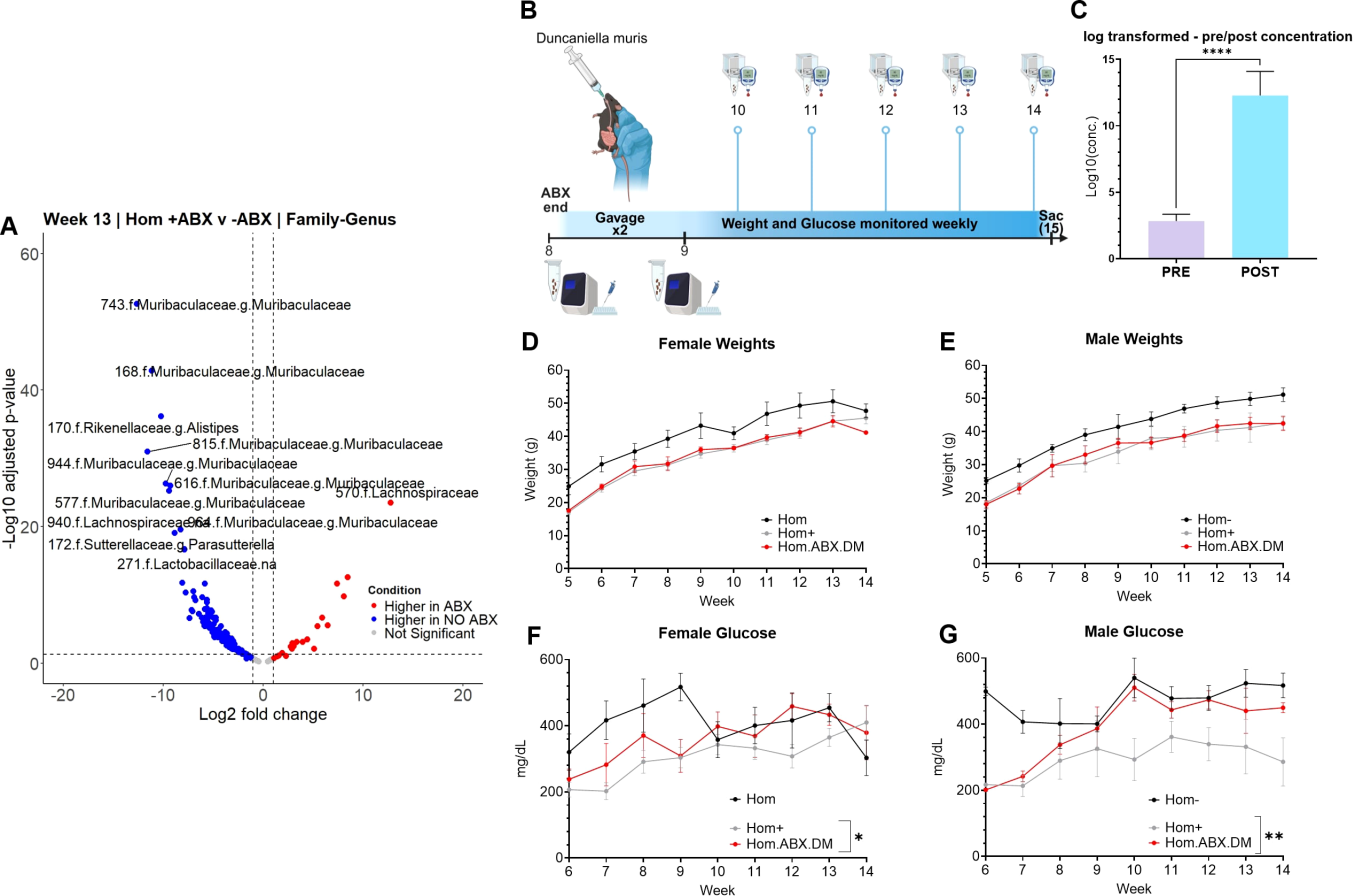
*Duncaniella muris* significantly increases blood glucose to pre-ABX treatment levels in Hom db/db mice. **(A)** DESeq2 at 13-weeks-old of treated versus untreated female and male Hom mice, using individual ASVs. *n* = 17. **(B)** Experimental schematic of supplementation experiment. **(C)** qPCR results of *D. muris* before and after gavage. Significance determined using Welch’s t-test. *n* = 4-8. **(D)** Female weights. Weights of Hom and Hom+ are repeated from **Figure 2A** Hom and Hom+ABX lines. Hom.ABX.DM *n* = 5 per timepoint. **(E)** Male weights. Weights of Hom and Hom+ are repeated from **Figure 2C** Hom and Hom+ABX lines. Hom.ABX.DM *n* = 4 per timepoint. **(F)** Female glucose. Glucose of Hom and Hom+ are repeated from **Figure 2B** Hom and Hom+ABX lines. Hom.ABX.DM *n* = 5 per timepoint. **(G)** Male glucose. Glucose of Hom and Hom+ are repeated from **Figure 2D** Hom and Hom+ABX lines. Hom.ABX.DM *n* = 4 per timepoint. [**(D–G)** significance reflects mean value across the full longitudinal window shown and was calculated using a mixed-effects Two-way ANOVA]. Significance values as follows: ns p ≥ 0.05, *p = 0.01 to 0.05, **p = 0.001 to 0.01, ***p = 0.0001 to 0.001, ****p < 0.0001.

At 13-weeks-old, the db/db phenotype is fully established, and the mice that were given ABX had five weeks to recover. This recovery period allows for comparative analysis between treated and untreated Hom mice to capture the differential abundances contributing to the phenotypic differences observed (**Figures 2A–D**). Taxa that distinguish treated from untreated Hom mice become further defined as the ABX-treated mice recover (**Supplementary Figures 7A–D**; **Supplementary Figures 8A,B**). During this recovery period, we observed that the reduced weight gain and glucose levels were sustained while the microbiome gradually adjusted. To investigate the specific bacterial taxa which might contribute to the obesity phenotype, we performed differential abundance analysis using DESeq2, comparing fecal samples from untreated Hom mice to Hom mice treated with ABX at 13-weeks-old (**Figure 5A**). DESeq2 analysis of the unique ASVs assigned to each sequence identified several *Muribaculaceae* genera as having a higher differential abundance in male and female homozygous mice at 13-weeks-old—separately, and when females and males were grouped together (**Figure 5A**; **Supplementary Figures 8A,B**). The sequences were ASVs 743, 168, 815, 944, 577, and 616 (MaAsLin2; 743 coef = 1.5, p < 0.0001; 168 coef = 1.7, p < 0.0001; 815 coef = 9.0, p < 0.0001; 944 coef = 2.2, p < 0.0001; 577 coef = 2.5, p < 0.0001; and 616 coef = 1.9, p < 0.0001); (**Figure 5A****;** **Supplementary Table 1**). When we compared week 13 data to week 5 data, we noticed that *Muribaculaceae* consistently dominated in untreated mice, though the specific ASVs changed as the mice aged (**Supplementary Figures 7A–D**; **Supplementary Figures 8A, B**). To refine our taxonomic resolution, we performed BLAST alignment of these ASV sequences against the NCBI microbe database. This analysis revealed high similarity across several sequences, with multiple alignments to *Duncaniella muris*. It is likely that these different ASVs originate from the same microbial species, but group separately due to sequencing variability. Plotting these ASVs separately as relative abundance indicates that ABX led to a broad reduction among these ASVs (**Supplementary Figure 9**; **Supplementary Table 1**). Specifically, ASV 743 had the strongest likely identity to *D. muris*, while 577 had a secondary alignment to *D. muris*. Since *D. muris* was the most robustly reduced taxon after antibiotic exposure and recovery, we hypothesized that its abundance could explain the lower weight and glucose levels in the antibiotic-treated Hom mice.

*D. muris* is one of the few cultured and identified species of the *Muribaculaceae* family, which dominates the normal murine microbiome (Lagkouvardos et al., 2019). *D. muris* was only recently described in Lagkouvardos et al., 2019, and thus, not much is known about the function of the bacteria in the microbiome. We hypothesized that gavaging Hom+ABX mice with *D. muris* would reverse the reduced weight gain phenotype that we had formerly observed back to untreated Hom conditions (**Figures 2A,C**). Following cessation of antibiotic treatment at 8-weeks-old, Hom mice received two oral gavages of cultured *D. muris* (10^7^ CFU), four days apart (**Figure 5B**). Fecal pellets collected before and after gavage showed a significant increase in *D. muris* DNA via qPCR, confirming successful colonization (**Figure 5C**). Contrary to our hypothesis, *D. muris* supplementation did not lead to any significant change in body weight in ABX-treated Hom mice of either sex (**Figures 5D, E**). On the other hand, *D. muris* significantly increased glucose levels in both female and male mice, with a more pronounced effect in males, effectively restoring glucose to pre-ABX levels (**Figures 5F ,G**). This suggests that the most differentially abundant taxa at week 13 may play an important role in glucose regulation. The lack of weight gain following *D. muris* supplementation supports a model in which microbial influences on body mass are established earlier in development, potentially preceding age 13 weeks. In contrast, glucose metabolism may remain sensitive to microbial cues later into life (Cox and Blaser, 2014; Schulfer et al., 2019; Schell and Carmody, 2025). Thus, we conclude that *D. muris* promotes hyperglycemia in the db/db mouse model.

### 3.6 *D. muris* is positively associated with *Bacteroidaceae*, and negatively associated with *Lachnospiraceae* and *Ruminococcaceae* in db/db mice

**Figure 6:**
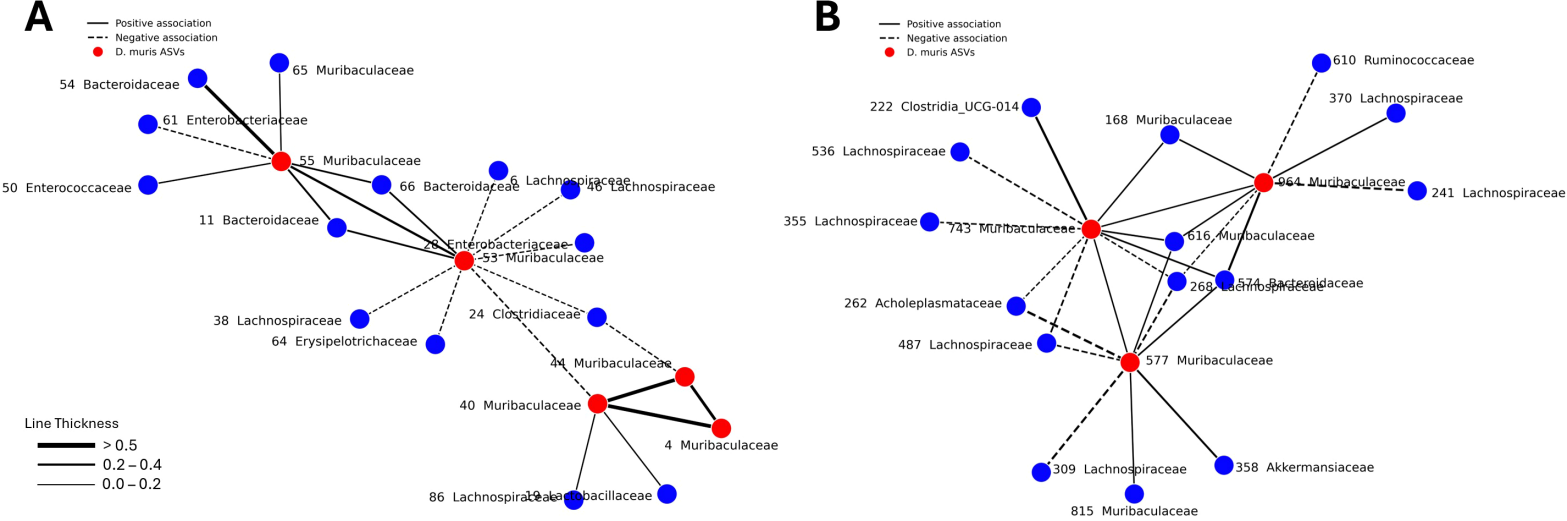
*D. muris* is positively associated with *Bacteroidaceae*, and negatively associated with *Lachnospiraceae* and *Ruminococcaceae* in db/db mice. **(A)** Co-abundance network of D. muris ASVs in Hom.ABX.DM male and female mice. Line thickness indicates association strength. This is a subset of the co-abundance network which can be found in full in **Supplementary Table 2**. *n* = 17. **(B)** Co-abundance network of D. muris ASVs in untreated Hom mice. Line thickness indicates association strength. This is a subset of the co-abundance network which can be found in full in **Supplementary Table 2**. *n* = 16.

Gut microbes do not function in isolation; individual taxa engage in complex relationships that shape community-level function. To investigate whether supplementation of *D. muris* into Hom+ABX mice restructured community composition in a manner that was associated with increased blood glucose, we constructed pairwise conditional association, correlational co-abundance networks from 16S ASV data for Hom.ABX.DM mice aged 10–13 weeks (**Supplementary Table 2**). Each node is labeled with the ASV identifier and corresponding bacterial family. Positive and negative associations are represented by solid and dashed edges, respectively, and in **Figures 6A, B** *D. muris* ASVs are highlighted in red.

In the Hom.ABX.DM dataset, BLAST alignment identified ASVs 55, 53, 40, 44, and 4 as closely matching *D. muris*. ASVs 55 and 53 exhibited consistent negative associations with 3 *Lachnospiraceae* ASVs and positive associations with 3 *Bacteroidaceae* ASVs, suggesting that *D. muris* colonization is associated with reciprocal shifts in these two families (**Figure 6A**). A single *Lachnospiraceae* ASV showed a positive association with ASV 40, which formed a separate triangular sub-network with ASVs 44 and 4 (**Figure 6A**). This cluster was negatively associated with the broader *D. muris* network. We interpret this sub-network as representing native host *D. muris* ASVs, because BLAST alignment of ASVs 40, 44, and 4 yielded lower sequence identity to the supplemented *D. muris* sequences than ASVs 55 and 53 (**Supplementary Table 1**). The positive *Lachnospiraceae* association within this sub-network may therefore reflect interactions with this native *D. muris* population already present in the host, rather than the supplemented strain.

To determine whether the co-abundance patterns observed in Hom.ABX.DM mice were recapitulated in untreated Hom mice, we created a co-abundance network for these mice aged 10–13 weeks (**Figure 6B**). Untreated Hom mice develop severe obesity and hyperglycemia, and we reasoned that finding similar microbial associations in these mice would help us identify which bacteria *D. muris* interacts with to promote hyperglycemia. In the untreated Hom dataset, *D. muris* was represented by ASVs 743, 964, and 577. These ASVs showed negative associations with 6 *Lachnospiraceae* ASVs and 1 *Ruminococcaceae* ASV, and a positive association with 1 *Bacteroidaceae* ASV that was shared across all 3 *D. muris* nodes (**Figure 6B****;** **Supplementary Table 2**). This matches the pattern we observed in Hom.ABX.DM mice (**Figure 6A**). The higher number of negatively associated *Lachnospiraceae* and *Ruminococcaceae* ASVs in the untreated Hom network is likely because the gut microbiome is more diverse in antibiotic-naive mice, meaning these bacterial families are more abundantly represented. We also detected a positive association between 1 *Lachnospiraceae* ASV and *D. muris* ASV 964 (**Figure 6B**; **Supplementary Table 2**). We attribute this to the fact that antibiotics were never used in these mice, so the niche was likely never cleared and some *Lachnospiraceae* members may still co-exist alongside *D. muris*. Thus, the fact that both networks generally show the same directional associations with *D. muris*, negative with *Lachnospiraceae* and *Ruminococcaceae* and positive with *Bacteroidaceae*, suggests that these relationships are consistent and not specific to one experimental condition.

## 4 Discussion

This study demonstrates that obesity in db/db mice requires not only genetic predisposition but also a permissive gut microbiome established during early life. Through perinatal antibiotic perturbation, we revealed that microbial perturbation reduced both weight gain and hyperglycemia. We identified *Duncaniella muris* as a key mediator of hyperglycemia in this genetic obesity model, establishing a direct link between a specific microbe and glucose dysregulation on standard chow.

Antibiotic treatment depleted *Muribaculaceae*, *Rikenellaceae*, *Lachnospiraceae*, and *Lactobacillaceae* in Hom mice. *Muribaculaceae* are gram-negative bacteria that attach to the mucus layer and produce SCFAs related to increased mucin release, reduced inflammation, and improved intestinal barrier function (You et al., 2022; Lagkouvardos et al., 2019). Research shows *Muribaculaceae* negatively correlate with obesity risk (Zhu et al., 2024; Mamun et al., 2025). While *Muribaculaceae* dominate the murine gut, humans predominantly harbor the functionally analogous *Bacteroidaceae*, which belongs to the same phylum, *Bacteroidota* (Zhu et al., 2024). *Bacteroidaceae* also function in the mucosal lining where O-glycan degradation and SCFA production contribute to intestinal barrier integrity (Pudlo et al., 2015; Ryan et al., 2020). Interestingly, novel, undefined pathobionts within *Muribaculaceae* have been implicated in supplementation experiments, in which a disrupted microbiome permits bacterial translocation to the pancreas, causing pancreatic inflammation and β-cell death (Yang et al., 2023). This β-cell death results in insulin-dependent diabetes, suggesting certain taxa may promote diabetes via the gut microbiome (Yang et al., 2023). Identifying *D. muris* as a causal driver of hyperglycemia but not weight gain provides a direct link between a defined microbe and a metabolic phenotype. *D. muris* is one of the few cultured *Muribaculaceae* species, and has a genome enriched in carbohydrate-active enzymes (Lagkouvardos et al., 2019). Experimental literature on *D. muris* is sparse; however, available studies indicate that its physiological effects in disease settings are context-dependent. An *in vitro* study showed that *D. muris* can produce SCFAs from exopolysaccharides derived from *Streptococcus salivarius*, suggesting that *D. muris* may have a metabolic impact depending on its microbial environment and the available polysaccharide substrates (Shimizu et al., 2025). Another study using a DSS colitis mouse model showed that *D. muris* colonization increased susceptibility to intestinal inflammation in specific-pathogen-free mice (Iljazovic et al., 2021). Increased intestinal inflammation was associated with reduced luminal acetate and decreased colonic IL-18, suggesting that *D. muris* may worsen intestinal inflammation in some settings by altering microbial metabolite production and epithelial immune signaling (Iljazovic et al., 2021). The mechanisms by which *D. muris* elevates glucose, however, remain elusive. Like *Akkermansia muciniphila*, which degrades mucin O-glycans and is associated with improved barrier integrity and glucose control, *D. muris* likely alters mucin turnover, SCFA composition, and downstream incretin or insulin signaling (Dao et al., 2016; Bakshani et al., 2025). The inflammatory impacts of these bacteria may indicate potential therapeutic targets for probiotics or FMT in obesity and diabetes treatment. Reducing the abundance of particular taxa could potentially lessen pancreatic inflammation and the progression to insulin-dependent diabetes. The data presented here cannot distinguish whether *Muribaculaceae* act through gastrointestinal metabolic mechanisms, or via other proinflammatory pathways to increase insulin resistance.

Host genetics influenced baseline microbiome composition, with Het and Hom animals clustering separately and differing in relative abundance of key families. Our findings parallel reports that ob/ob and db/db mice harbor communities distinct from lean controls (Yang et al., 2019). *Erysipelotrichaceae*, enriched in Hom+ABX mice, have been associated with inflammation and obesity (Kaakoush, 2015; Zhang et al., 2023; Truax et al., 2018). Conversely, *Lachnospiraceae* and *Lactobacillaceae*, elevated in Het+ABX mice, include butyrate producers which are associated with reduced weight and lower inflammation (Li et al., 2023; Heczko et al., 2024). These contrasting profiles may explain the stronger antibiotic impact on weight in Hom than Het mice. Notably, Clostridia_UCG-014 enriched in hyperglycemic, Hom, mice positively correlates with HDL cholesterol in overweight patients with early-onset type 2 diabetes, highlighting the db/db model’s value for identifying obesity-linked taxa (Huang et al., 2025).

Sex-specific baseline differences may underlie differing antibiotic responses in Het mice. Female Hets showed lower *Muribaculaceae* and higher *Lactobacillaceae* than males, while males exhibited increased *Erysipelotrichaceae*. This may explain the decreased weight in Het+ABX male mice compared to their untreated counterparts, which was not observed in female mice. These patterns align with reports of sex-biased gut community distributions in mice (McGee and Huttenhower, 2021; Kim et al., 2020; Wu et al., 2022). Male Het+ABX mice—but not females—displayed reduced weight gain, suggesting the male Het microbiome harbors antibiotic-sensitive obesogenic taxa, whereas female microbiomes may be buffered by *Lactobacillaceae*-enriched communities. The compositional differences may carry direct metabolic consequences. *Lactobacillaceae*-enriched communities could influence inflammatory tone and lactate-SCFA cross-feeding, while the preservation of SCFA-associated metabolic signaling may help buffer female Het mice against antibiotic-induced weight changes despite broader community disruption (Heczko et al., 2024; Yadav et al., 2013). This interpretation is consistent with experimental evidence that antibiotic exposure produces sex-dependent changes in gut microbial composition, SCFA metabolism, and amino acid metabolism in mice, suggesting that males and females may differ not only in which taxa are remodeled, but also in the metabolic outputs generated after perturbation (Gao et al., 2019). Although cohousing likely limited how far Het and Hom microbiomes could diverge at baseline, we still observed significant genotype separation and clear genotype- and sex-specific responses to antibiotics, indicating that host genetics and sex shape how shared microbial pools are remodeled under perturbation (Ericsson et al., 2017; Vrieze et al., 2012; Ridaura et al., 2013; Daoust et al., 2023).

Following antibiotic cessation, Hom mice became dominated by *Akkermansiaceae* and *Bacteroidaceae*, and subsequent supplementation with *D. muris* established a correlational co-abundance network characterized by a positive *D. muris*-*Bacteroidaceae* association, alongside suppression of *Lachnospiraceae* and *Ruminococcaceae*. This community configuration closely resembles the *Bacteroides* 2 enterotype, a gut microbial state associated with obesity that is defined by the suppression of *Lachnospiraceae* and *Ruminococcaceae* (Molinaro et al., 2020). Molinaro et al. further showed that the *Bacteroides* 2 enterotype is associated with elevated serum imidazole propionate (ImP) and enrichment of the histidine-to-ImP pathway genes *hutH* and *urdA*, where HutH converts histidine to urocanate and UrdA converts urocanate to ImP. Several *Bacteroidaceae* present in the *Bacteroides* 2 enterotype carry the *urdA* gene, and examination of the *D. muris* strain used in our supplementation experiments revealed that its genome encodes *hutH*, the first enzyme in this pathway (Schober et al., 2025). Once ImP enters circulation, it impairs insulin signaling by activating the p38γ/p62/mTORC1 pathway, which inhibits insulin receptor substrate signaling and results in insulin resistance and impaired blood glucose control. Notably, histidine is a standard component of the mouse chow used in this study, present at 6 milligrams per gram of chow (LabDiet, 2025). Given that Hom+ABX female and male mice consumed approximately 9 and 12 grams of chow per day, respectively, they were ingesting roughly 54 and 72 mg of histidine daily, providing a plausible endogenous substrate for ImP production (**Figure 2F**). This difference in histidine consumption may also help explain the more rapid progression to hyperglycemia observed in males compared to females following *D. muris* supplementation. The concurrent suppression of *Lachnospiraceae* and *Ruminococcaceae* within this community configuration may also reduce butyrate availability, providing a parallel metabolite-mediated mechanism through which microbial remodeling could impair glucose control. Butyrate has been shown to improve metabolic health in a microbiota-dependent manner and is associated with insulin homeostasis in humans, while probiotic-induced increases in butyrate can stimulate glucagon-like peptide-1 secretion and improve glucose tolerance in mice (Li et al., 2023; Cui et al., 2022; Yadav et al., 2013). Thus, the *D. muris*-associated suppression of butyrate-linked taxa may converge with the proposed ImP pathway by reducing metabolite signals that normally support insulin sensitivity, incretin signaling, and metabolic homeostasis. Direct measurement of fecal and circulating SCFAs, alongside ImP and histidine-pathway activity, will be required to determine whether these pathways act independently or together to promote hyperglycemia in this model. Together, these findings suggest that *D. muris* may promote hyperglycemia in part by contributing to a *Bacteroides* 2-like microbial state that facilitates ImP-mediated impairment of insulin signaling, though direct measurement of ImP and pathway activity will be needed to confirm this mechanism.

### 4.1 Limitations

Our study has several limitations. Mouse numbers per group were limited by breeding ratios and the demands of maintaining sex-, genotype-, and treatment-matched cohorts across multiple experimental conditions. Though the consistency of our findings across sexes and timepoints supports their validity, future studies employing more breeding pairs would further strengthen these findings. We used 16S rRNA sequencing rather than shotgun metagenomics, limiting taxonomic resolution and preventing direct inference of microbial pathways. Quantitative PCR would have further helped to validate the reduction of particular microbial groups observed in the relative abundance, DESeq2, and MaAsLin2 data. Cohousing may have dampened genotype-specific differences and introduced cage effects. Additionally, antibiotic-exposed breeding pairs produced one to four surviving litters, and because parental duration of antibiotic exposure differed across successive litters, we cannot exclude litter order or cumulative parental antibiotic exposure as potential contributors to microbiome or metabolic variation. Antibiotics were administered ad libitum, so dosing may vary between mice. We administered a cocktail of antibiotics; thus, we cannot determine which individual antibiotics drove specific microbial or metabolic effects. Our gavage experiment tested *D. muris* at one timepoint; thus, we cannot tell if administering *D. muris* earlier or later would have impacted obesity. Thus, we can say that *D. muris* impacts hyperglycemia, but we cannot conclude positively or negatively whether it impacts weight. Furthermore, supplementation of *D. muris* and a *Bacteroidaceae* consortium into gnotobiotic mice, both independently and in combination, would be required to determine whether their interaction is necessary for elevated blood glucose or whether *D. muris* acts alone. Further studies are needed to measure barrier integrity, SCFAs, bile acids, and host signaling. These future studies combining shotgun metagenomics, gnotobiotic models, and targeted metabolomics will be essential to define causal microbial networks driving weight gain. Despite these limitations, *D. muris* supplementation restored hyperglycemia while early-life antibiotics durably reprogrammed metabolism, demonstrating that the gut microbiome actively drives genetic obesity rather than passively reflecting it.

## Data Availability

The datasets presented in this study can be found in online repositories. The names of the repository/repositories and accession number(s) can be found below: https://www.ncbi.nlm.nih.gov/sra/PRJNA1366719.
