# Supplementary material for: Perinatal antibiotics in a db/db mouse model impact obesity and hyperglycemia in a microbiome-dependent manner
Source: Front Microbiomes. 2026 Jul 27;5:1750320. doi: 10.3389/frmbi.2026.1750320 (PMC13454812; doi:10.3389/frmbi.2026.1750320)
Supplement: Supplementary file 1 [file Supplementaryfile1.docx]

**Supplementary Figure S1.** Bacterial load at 5 weeks old shown as copies of the 16S gene per milligram of fecal sample. Hom indicates homozygous (db/db) mice. Het indicates heterozygous (db/+) mice. +ABX indicates antibiotic-treated mice. Untreated mice indicated as only Het or Hom label. *n* = 5 per group.
