# Supplementary material for: Perinatal antibiotics in a db/db mouse model impact obesity and hyperglycemia in a microbiome-dependent manner
Source: Front Microbiomes. 2026 Jul 27;5:1750320. doi: 10.3389/frmbi.2026.1750320 (PMC13454812; doi:10.3389/frmbi.2026.1750320)
Supplement: Supplementary file 2 [file Supplementaryfile10.docx]

| **ASV** | **Sequence** | **Top 3 Identities** |
| --- | --- | --- |
| 4.f.Muribaculaceae.g.Muribaculaceae | GCGAGCGTTATCCGGATTTATTGGGTTTAAAGGGTGCGCAGGCGGAATGTCAAGTCAGCGGTAAAATTTCGGGGCTCAACCCCGTCGTGCCGTTGAAACTGGCGTTCTTGAGTGAGCGAGAAGTATGCGGAATGCGTGGTGTAGCGGTGAAATGCATAGATATCACGCAGAACTCCGATTGCGAAGGCAGCATACCGGCGCTCAACTGACGCTCATGCACGAAAGCGTAGGT | Duncaniella dubosii, 99.57%; Muribaculum intestinale, 95.69%; Duncaniella muris, 94.83% |
| 40.f.Muribaculaceae.g.Muribaculaceae | GCGAGCGTTATCCGGATTTATTGGGTTTAAAGGGTGCGCAGGCGGAATGTCAAGTCAGCGGTAAAATTTCGGGGCTCAACCCCGTCGTGCCGTTGAAACTGGCGTTCTTGAGTGAGCGAGAAGTATGCGGAATGCGTGGTGTAGCGGTGAAATGCATAGATATCACGCAGAACTCCGATTGCGAAGGCAGCATACCGGCGCTCAACTGACGCTCATGCACGAAAGCGTGGGT | Duncaniella dubosii, 100.00%;  Muribaculum intestinale, 96.12%;  Duncaniella muris, 95.26% |
| 44.f.Muribaculaceae.g.Muribaculaceae | GCGAGCGTTATCCGGATTTATTGGGTTTAAAGGGTGCGCAGGCGGAATGTCAAGTCAGCGGTAAAATTTCGGGGCTCAACTCCGTCGTGCCGTTGAAACTGGCGTTCTTGAGTGAGCGAGAAGTATGCGGAATGCGTGGTGTAGCGGTGAAATGCATAGATATCACGCAGAACTCCGATTGCGAAGGCAGCATACCGGCGCTCAACTGACGCTCATGCACGAAAGCGTGGGT | Duncaniella dubosii, 99.57%;  Muribaculum intestinale, 95.69%;  Duncaniella muris, 94.83% |
| 53.f.Muribaculaceae.g.Muribaculaceae | GCGAGCGTTATCCGGATTTATTGGGTTTAAAGGGTGCGTAGGCGGTATGTCAAGTCAGCGGTAAAATTGAGAGGCTCAACTTCTTCGAGCCGTTGAAACTGGCATACTTGAGTGGGCGAGAAGTATGCGGAATGCGTGGTGTAGCGGTGAAATGCATAGATATCACGCAGAACTCCGATTGCGAAGGCAGCATACCGGCGCCCAACTGACGCTGAAGCACGAAAGCGTGGGG | Heminiphilus faecis, 94.83%;  Duncaniella muris, 94.37%;  Muribaculum intestinale, 93.94% |
| 55.f.Muribaculaceae.g.Muribaculaceae | GCGAGCGTTATCCGGATTTATTGGGTTTAAAGGGTGCGTAGGCGGTATGTCAAGTCAGCGGTAAAATTGAGAGGCTCAACCTCTTCGAGCCGTTGAAACTGGCATACTTGAGTGGGCGAGAAGTATGCGGAATGCGTGGTGTAGCGGTGAAATGCATAGATATCACGCAGAACTCCGATTGCGAAGGCAGCATACCGGCGCCCAACTGACGCTGAAGCACGAAAGCGTGGGG | Heminiphilus faecis, 95.26%;  Duncaniella muris, 94.81%;  Muribaculum intestinale, 94.37% |
| 254.f.Clostridiaceae.g.Clostridium_sensu_stricto_1 | GCGAGCGTTGTCCGGATTTACTGGGCGTAAAGGGAGCGTAGGCGGACTTTTAAGTGAGATGTGAAATACTCGGGCTCAACTTGAGTGCTGCATTTCAAACTGGAAGTCTAGAGTGCAGGAGAGGAGAATGGAATTCCTAGTGTAGCGGTGAAATGCGTAGAGATTAGGAAGAACACCAGTGGCGAAGGCGATTCTCTGGACTGTAACTGACGCTGAGGCTCGAAAGCGTGGGG | Clostridium celatum, 99.14%, Clostridium cuniculi, 99.14%; Clostridium saudiense, 99.14% |
| 106.f.Erysipelotrichaceae.g.Dubosiella | GCGAGCGTTATCCGGAATGATTGGGCGTAAAGGGTGCGCAGGCGGCCGGGCAAGTCCGCAGTAAAAACTGGAGGCTCAACCTTCAGGGGCTGCGGAAACTGTCCGGCTGGAGAGCAGGAGAGGACGGTGGAACTCCATGTGTAGCGGTAAAATGCGTAGATATATGGAAGAACACCGGTGGCGAAGGCGGCCGTCTGGCCTGCATCTGACGCTGAGGCACGAAAGCGTGGGG | Dubosiella newyorkensis, 100%; Flagellimonas ochraceae, 88.09%; Dubosiella muris, 87.93% |
| 441.f.Lachnospiraceae.na | GCAAGCGTTATCCGGATTTACTGGGTGTAAAGGGAGCGTAGACGGTAATGTAAGTCTGATGTGAAAGCCCGGGGCTCAACCCCGGGACTGCATTGGAAACTATGTAACTAGAGTACCGGAGAGGTAAGCGGAATTCCTAGTGTAGCGGTGAAATGCGTAGATATTAGGAGGAACACCAGTGGCGAAGGCGGCTTACTGGACGGAAACTGACGTTGAGGCTCGAAAGCGTGGGG | Coprococcus phoceensis, 97.00%; Faecalicatena contorta, 96.57%; Natronogracilivirga saccharolytica, 96.15% |
| 675.f.Muribaculaceae.g.Muribaculaceae | GCGAGCGTTATCCGGATTTATTGGGTTTAAAGGGTGCGTAGGCGGCCTGCCAAGTCAGCGGTAAAATTGCGGGGCTCAACCCCGTACAGCCGTTGAAACTGCCGGGCTCGAGTGGGCGAGAAGTATGCGGAATGCGTGGTGTAGCGGTGAAATGCATAGATATCACGCAGAACCCCGATTGCGAAGGCAGCATACCGGCGCCCTACTGACGCTGAGGCACGAAAGTGCGGGG | Clostridium cuniculi, 100.00%; Heminiphilus faecis, 93.07%; Muribaculum gordoncarteri, 93.36% |
| 750.f.Muribaculaceae.g.Muribaculaceae | GCGAGCGTTATCCGGATTTATTGGGTTTAAAGGGTGCGTAGGCGGCCTGCCAAGTCAGCGGTAAAATTGCGGGGCTCAACCCCGTACAGCCGTTGAAACTGCCGGGCTCGAGTGGGCGAGAAGTATGCGGAATGCGTGGTGTAGCGGTGAAATGCATAGATATCACGCAGAACCCCGATTGCGAAGGCAGCATACCGGCGCCCGACTGACGCTGAGGCACGAAAGTGCGGGG | Clostridium cuniculi, 99.57%; Heminiphilus faecis, 93.07%; Muribaculum gordoncarteri, 93.36 |
| 932.f.Lachnospiraceae.na | GCAAGCGTTATCCGGATTTACTGGGTGTAAAGGGAGCGTAGGCGGCCATGCAAGTCAGAAGTGAAAACCCGGGGCTCAACCCTGGGAGTGCTTTTGAAACTGTGCGGCTAGAGTGTCGGAGGGGTAAGTGGAATTCCTAGTGTAGCGGTGAAATGCGTAGATATTAGGAGGAACACCAGTGGCGAAGGCGGCTTACTGGACGATGACTGACGCTGAGGCTCGAAAGCGTGGGG | Muricomes intestini, 95.28%; Eubacterium segne, 94.87%; Eubacterium ventriosum, 94.87% |
| 91.f.Lachnospiraceae.na | GCAAGCGTTATCCGGATTTACTGGGTGTAAAGGGAGCGTAGGCGGCCACGCAAGTCAGAAGTGAAAACCCGGGGCTCAACCCTGGGAGTGCTTTTGAAACTGTGCGGCTAGAGTGTCGGAGGGGTAAGTGGAATTCCTAGTGTAGCGGTGAAATGCGTAGATATTAGGAGGAACACCAGTGGCGAAGGCGGCTTACTGGACGATGACTGACGCTGAGGCTCGAAAGCGTGGGG | Muricomes intestini, 94.85%; Eubacterium segne, 94.42%; Eubacterium ventriosum, 94.42% |
| 743.f.Muribaculaceae.g.Muribaculaceae | GCGAGCGTTATCCGGATTTATTGGGTTTAAAGGGTGCGCAGGCGGGCTGTCAAGTCAGCGGTAAAATTGAGAGGCTCAACCTCTTCGAGCCGTTGAAACTGGCGGTCTTGAGTGAGCGAGAAGTACGCGGAATGCGTGGTGTAGCGGTGAAATGCATAGATATCACGCAGAACTCCGATTGCGAAGGCAGCGTACCGGCGCTCAACTGACGCTCATGCACGAAAGCGTGGGT | Duncaniella muris, 95.69%; Duncaniella dubosii, 95.26%; Paramuribaculum intestinale, 93.16% |
| 168.f.Muribaculaceae.g.Muribaculaceae | GCGAGCGTTATCCGGATTTATTGGGTTTAAAGGGTGCGCAGGCGGACTCTCAAGTCAGCGGTCAAATCGCGGGGCTCAACCCCGTTCCGCCGTTGAAACTGGGAGCCTTGAGTGCGCGAGAAGTAGGCGGAATGCGTGGTGTAGCGGTGAAATGCATAGATATCACGCAGAACTCCGATTGCGAAGGCAGCCTACCGGCGCGCAACTGACGCTCATGCACGAAAGCGTGGGT | Duncaniella freteri, 94.83%; Duncaniella muricolitica, 94.40%; Duncaniella dubosii, 93.10% |
| 815.f.Muribaculaceae.g.Muribaculaceae | GCGAGCGTTATCCGGATTTATTGGGTTTAAAGGGTGCGTAGGCGGACTGTCAAGTCAGCGGTAAAATTGAGAGGCTCAACCTCTTCCCGCCGTTGAAACTGGTGGTCTTGAGTGGATGAGAAGTACGCGGAATGCGTGGTGTAGCGGTGAAATGCATAGATATCACGCAGAACTCCGATTGCGAAGGCAGCGTACCGGCATCCAACTGACGCTGAGGCACGAAAGCGTGGGG | Muribaculum gordoncarteri, 93.51%; Heminiphilus faecis, 92.64%; Paramuribaculum intestinale, 92.64% |
| 944.f.Muribaculaceae.g.Muribaculaceae | GCGAGCGTTATCCGGATTTATTGGGTTTAAAGGGTGCGTAGGCGGTCCGTTAAGTCAGCGGTAAAATTGCGGGGCTCAACCCCGTCGAGCCGTTGAAACTGGCAGACTTGAGTTGGCGAGAAGTACGCGGAATGCGCGGTGTAGCGGTGAAATGCATAGATATCGCGCAGAACTCCGATTGCGAAGGCAGCGTACCGGCGCCAGACTGACGCTGAGGCACGAAAGCGTGGGG | Sangeribacter muris, 100.00%; Paramuribaculum intestinale, 93.94%; Lepagella muris, 92.64% |
| 616.f.Muribaculaceae.g.Muribaculaceae | GCGAGCGTTATCCGGATTTATTGGGTTTAAAGGGTGCGTAGGCGGGCTGATAAGTCAGCGGTTAAAATGCGGTGCTCAACGCCGTAGTGCCGTTGAAACTGTCGGTCTTGAGTGAGCGAGAAGTATGCGGAATGCGTGGTGTAGCGGTGAAATGCATAGATATCACGCAGAACTCCGATTGCGAAGGCAGCATACCGGCGCTCAACTGACGCTGAAGCACGAAAGCGTGGGT | Muribaculum intestinale, 93.99%; Duncaniella dubosii, 93.53%; Muribaculum gordoncarteri, 93.16% |
| 185.f.Muribaculaceae.g.Muribaculaceae | TCAAGCGTTATCCGGATTTATTGGGTTTAAAGGGTGCGCAGGCGGGCTGTCAAGTCAGCGGTAAAATTGAGAGGCTCAACCTCTTCGAGCCGTTGAAACTGGCGGTCTTGAGTGAGCGAGAAGTACGCGGAATGCGTGGTGTAGCGGTGAAATGCGTAGATATTAGGAGGAACACCGATGGCGAAGGCAGCCTCCTGGGACATAACTGACGCTCAGGCACGAAAGCGTGGGG | Duncaniella muris, 89.47%; Duncaniella dubosii, 89.04%; Sangeribacter muris, 87.39% |
| 577.f.Muribaculaceae.g.Muribaculaceae | GCGAGCGTTATCCGGATTTATTGGGTTTAAAGGGTGCGTAGGCGGTATGTCAAGTCAGCGGTAAAATTGAGAGGCTCAACCTCTTCGAGCCGTTGAAACTGGCATACTTGAGTGGGCGAGAAGTATGCGGAATGCGTGGTGTAGCGGTGAAATGCATAGATATCACGCAGAACTCCGATTGCGAAGGCAGCATACCGGCGCCCAACTGACGCTGAAGCACGAAAGCGTGGGG | Heminiphilus faecis, 95.26%; Duncaniella muris, 94.81%; Muribaculum intestinale, 94.37% |
| 773.f.Muribaculaceae.g.Muribaculaceae | GCGAGCGTTGTCCGGAATTACTGGGTGTAAAGGGAGCGTAGGCGGGATGTCAAGTCAGAAGTGAAAATTATGGGCTCAACTCATAACCTGCTTTTGAAACTGATATTCTTGAGTGAGCGAGAAGTACGCGGAATGCGTGGTGTAGCGGTGAAATGCATAGATATCACGCAGAACTCCGATTGCGAAGGCAGCGTACCGGCGCTCAACTGACGCTCATGCACGAAAGCGTGGGT | Duncaniella dubosii, 88.84%; Muribaculum intestinale, 88.41%; Duncaniella muris, 88.09% |
| 964.f.Muribaculaceae.g.Muribaculaceae | GCGAGCGTTATCCGGATTTATTGGGTTTAAAGGGTGCGTAGGCGGACAGTTAAGTCAGCGGTAAAATTGAGAGGCTCAACCTCTTCCCGCCGTTGAAACTGATTGTCTTGAGTGGGCGAGAAGTATGCGGAATGCGTGGTGTAGCGGTGAAATGCATAGATATCACGCAGAACTCCGATTGCGAAGGCAGCATACCGGCGCCCAACTGACGCTGAAGCACGAAAGCGTGGGT | Muribaculum gordoncarteri, 95.69%; Paramuribaculum intestinale, 94.40%; Duncaniella muris, 94.42% |

**Supplementary Table 1.** | ASV with sequence and top 3 alignments (using NCBI BLAST).
