# Supplementary material for: Perinatal antibiotics in a db/db mouse model impact obesity and hyperglycemia in a microbiome-dependent manner
Source: Front Microbiomes. 2026 Jul 27;5:1750320. doi: 10.3389/frmbi.2026.1750320 (PMC13454812; doi:10.3389/frmbi.2026.1750320)
Supplement: Supplementary file 3 [file Supplementaryfile11.docx]

| node1 | node2 | rho | sign |
| --- | --- | --- | --- |
| HOM.ABX.DM MICE | | | |
| 74.f.Lachnospiraceae | 86.f.Lachnospiraceae | 0.630114 | positive |
| 40.f.Muribaculaceae.g.Muribaculaceae | 4.f.Muribaculaceae.g.Muribaculaceae | 0.512023 | positive |
| 40.f.Muribaculaceae.g.Muribaculaceae | 44.f.Muribaculaceae.g.Muribaculaceae | 0.498442 | positive |
| 33.f.Akkermansiaceae.g.Akkermansia | 41.f.Akkermansiaceae.g.Akkermansia | 0.460786 | positive |
| 26.f.Akkermansiaceae.g.Akkermansia | 33.f.Akkermansiaceae.g.Akkermansia | 0.453315 | positive |
| 54.f.Bacteroidaceae.g.Bacteroides | 55.f.Muribaculaceae.g.Muribaculaceae | 0.448192 | positive |
| 80.f.Lachnospiraceae.g.A2 | 13.f.Lachnospiraceae | 0.4406 | positive |
| 62.f.Enterobacteriaceae | 28.f.Enterobacteriaceae | 0.43732 | positive |
| 24.f.Clostridiaceae.g.Clostridium_sensu_stricto_1 | 73.f.Staphylococcaceae.g.Staphylococcaceae | 0.397479 | positive |
| 49.f.Staphylococcaceae.g.Staphylococcus | 73.f.Staphylococcaceae.g.Staphylococcaceae | 0.367852 | positive |
| 64.f.Erysipelotrichaceae.g.Turicibacter | 18.f.Erysipelotrichaceae.g.Turicibacter | 0.365435 | positive |
| 46.f.Lachnospiraceae.g.Lachnospiraceae_NK4A136_group | 47.f.Lachnospiraceae.g.Lachnospiraceae_NK4A136_group | 0.358588 | positive |
| 4.f.Muribaculaceae.g.Muribaculaceae | 44.f.Muribaculaceae.g.Muribaculaceae | 0.353711 | positive |
| 79.f.Propionibacteriaceae.g.Cutibacterium | 18.f.Erysipelotrichaceae.g.Turicibacter | 0.347246 | positive |
| 75.f.Lachnospiraceae.g.Lachnospiraceae_NK4A136_group | 46.f.Lachnospiraceae.g.Lachnospiraceae_NK4A136_group | 0.34542 | positive |
| 19.f.Lactobacillaceae.g.HT002 | 64.f.Erysipelotrichaceae.g.Turicibacter | 0.344187 | positive |
| 78.f.Paenibacillaceae.g.Paenibacillus | 36.f.Butyricicoccaceae.g.Butyricicoccus | 0.337285 | positive |
| 26.f.Akkermansiaceae.g.Akkermansia | 41.f.Akkermansiaceae.g.Akkermansia | 0.336465 | positive |
| 6.f.Lachnospiraceae.g.Lachnospiraceae_UCG-001 | 5.f.Lachnospiraceae.g.Lachnospiraceae_NK4A136_group | 0.329107 | positive |
| 35.f.Muribaculaceae.g.Muribaculaceae | 65.f.Muribaculaceae.g.Muribaculaceae | 0.328448 | positive |
| 10.f.Enterobacteriaceae | 7.f.Erysipelotrichaceae.g.Turicibacter | -0.32432 | negative |
| 43.f.Clostridiaceae.g.Clostridium_sensu_stricto_1 | 36.f.Butyricicoccaceae.g.Butyricicoccus | 0.311861 | positive |
| 75.f.Lachnospiraceae.g.Lachnospiraceae_NK4A136_group | 47.f.Lachnospiraceae.g.Lachnospiraceae_NK4A136_group | 0.309184 | positive |
| 10.f.Enterobacteriaceae | 74.f.Lachnospiraceae | 0.303618 | positive |
| 32.f.Monoglobaceae.g.Monoglobus | 29.f.Lachnospiraceae | 0.301119 | positive |
| 61.f.Enterobacteriaceae | 49.f.Staphylococcaceae.g.Staphylococcus | 0.299371 | positive |
| 22.f.Lactobacillaceae.g.HT002 | 8.f.Lactobacillaceae.g.HT002 | 0.297207 | positive |
| 28.f.Enterobacteriaceae | 72.f.Lachnospiraceae.g.Lachnospiraceae_NK4A136_group | -0.29504 | negative |
| 38.f.Lachnospiraceae.g.Lachnospiraceae_UCG-001 | 60.f.Lachnospiraceae.g.Lachnospiraceae_UCG-001 | 0.272085 | positive |
| 23.o.Lactobacillales | 19.f.Lactobacillaceae.g.HT002 | 0.267341 | positive |
| 78.f.Paenibacillaceae.g.Paenibacillus | 8.f.Lactobacillaceae.g.HT002 | 0.264677 | positive |
| 38.f.Lachnospiraceae.g.Lachnospiraceae_UCG-001 | 68.f.Lachnospiraceae.g.Lachnospiraceae_UCG-001 | 0.264545 | positive |
| 51.f.Lachnospiraceae.g.Lachnospiraceae_NK4A136_group | 5.f.Lachnospiraceae.g.Lachnospiraceae_NK4A136_group | 0.261936 | positive |
| 43.f.Clostridiaceae.g.Clostridium_sensu_stricto_1 | 5.f.Lachnospiraceae.g.Lachnospiraceae_NK4A136_group | 0.2569 | positive |
| 23.o.Lactobacillales | 8.f.Lactobacillaceae.g.HT002 | -0.25654 | negative |
| 58.f.Lachnospiraceae | 69.f.Lachnospiraceae | 0.255311 | positive |
| 27.f.Lachnospiraceae | 58.f.Lachnospiraceae | 0.25289 | positive |
| 55.f.Muribaculaceae.g.Muribaculaceae | 53.f.Muribaculaceae.g.Muribaculaceae | 0.249 | positive |
| 27.f.Lachnospiraceae | 69.f.Lachnospiraceae | 0.241823 | positive |
| 79.f.Propionibacteriaceae.g.Cutibacterium | 8.f.Lactobacillaceae.g.HT002 | 0.238097 | positive |
| 54.f.Bacteroidaceae.g.Bacteroides | 65.f.Muribaculaceae.g.Muribaculaceae | 0.237022 | positive |
| 32.f.Monoglobaceae.g.Monoglobus | 3.f.Lachnospiraceae.g.Roseburia | 0.234741 | positive |
| 62.f.Enterobacteriaceae | 36.f.Butyricicoccaceae.g.Butyricicoccus | 0.23459 | positive |
| 70.f.Clostridiaceae.g.Clostridium_sensu_stricto_1 | 43.f.Clostridiaceae.g.Clostridium_sensu_stricto_1 | 0.219323 | positive |
| 66.f.Bacteroidaceae.g.Bacteroides | 54.f.Bacteroidaceae.g.Bacteroides | 0.211605 | positive |
| 11.f.Bacteroidaceae.g.Bacteroides | 54.f.Bacteroidaceae.g.Bacteroides | 0.21106 | positive |
| 68.f.Lachnospiraceae.g.Lachnospiraceae_UCG-001 | 60.f.Lachnospiraceae.g.Lachnospiraceae_UCG-001 | 0.210986 | positive |
| 19.f.Lactobacillaceae.g.HT002 | 74.f.Lachnospiraceae | 0.210597 | positive |
| 22.f.Lactobacillaceae.g.HT002 | 36.f.Butyricicoccaceae.g.Butyricicoccus | 0.209811 | positive |
| 79.f.Propionibacteriaceae.g.Cutibacterium | 7.f.Erysipelotrichaceae.g.Turicibacter | 0.207615 | positive |
| 49.f.Staphylococcaceae.g.Staphylococcus | 33.f.Akkermansiaceae.g.Akkermansia | -0.20694 | negative |
| 64.f.Erysipelotrichaceae.g.Turicibacter | 7.f.Erysipelotrichaceae.g.Turicibacter | 0.203952 | positive |
| 38.f.Lachnospiraceae.g.Lachnospiraceae_UCG-001 | 59.f.Lachnospiraceae | 0.200679 | positive |
| 24.f.Clostridiaceae.g.Clostridium_sensu_stricto_1 | 78.f.Paenibacillaceae.g.Paenibacillus | -0.2001 | negative |
| 6.f.Lachnospiraceae.g.Lachnospiraceae_UCG-001 | 51.f.Lachnospiraceae.g.Lachnospiraceae_NK4A136_group | 0.19977 | positive |
| 23.o.Lactobacillales | 74.f.Lachnospiraceae | 0.19922 | positive |
| 77.f.Lachnospiraceae | 27.f.Lachnospiraceae | 0.196884 | positive |
| 70.f.Clostridiaceae.g.Clostridium_sensu_stricto_1 | 46.f.Lachnospiraceae.g.Lachnospiraceae_NK4A136_group | -0.19659 | negative |
| 3.f.Lachnospiraceae.g.Roseburia | 29.f.Lachnospiraceae | 0.191592 | positive |
| 28.f.Enterobacteriaceae | 36.f.Butyricicoccaceae.g.Butyricicoccus | 0.18936 | positive |
| 43.f.Clostridiaceae.g.Clostridium_sensu_stricto_1 | 51.f.Lachnospiraceae.g.Lachnospiraceae_NK4A136_group | 0.187892 | positive |
| 23.o.Lactobacillales | 64.f.Erysipelotrichaceae.g.Turicibacter | 0.187135 | positive |
| 77.f.Lachnospiraceae | 58.f.Lachnospiraceae | 0.181761 | positive |
| 79.f.Propionibacteriaceae.g.Cutibacterium | 62.f.Enterobacteriaceae | -0.18058 | negative |
| 13.f.Lachnospiraceae | 29.f.Lachnospiraceae | 0.180106 | positive |
| 32.f.Monoglobaceae.g.Monoglobus | 13.f.Lachnospiraceae | 0.178863 | positive |
| 61.f.Enterobacteriaceae | 19.f.Lactobacillaceae.g.HT002 | -0.17853 | negative |
| 50.f.Enterococcaceae | 73.f.Staphylococcaceae.g.Staphylococcaceae | 0.176714 | positive |
| 61.f.Enterobacteriaceae | 50.f.Enterococcaceae | -0.17474 | negative |
| 23.o.Lactobacillales | 33.f.Akkermansiaceae.g.Akkermansia | 0.174267 | positive |
| 11.f.Bacteroidaceae.g.Bacteroides | 55.f.Muribaculaceae.g.Muribaculaceae | 0.17321 | positive |
| 78.f.Paenibacillaceae.g.Paenibacillus | 5.f.Lachnospiraceae.g.Lachnospiraceae_NK4A136_group | -0.17249 | negative |
| 66.f.Bacteroidaceae.g.Bacteroides | 11.f.Bacteroidaceae.g.Bacteroides | 0.170145 | positive |
| 43.f.Clostridiaceae.g.Clostridium_sensu_stricto_1 | 72.f.Lachnospiraceae.g.Lachnospiraceae_NK4A136_group | 0.169674 | positive |
| 28.f.Enterobacteriaceae | 19.f.Lactobacillaceae.g.HT002 | 0.165922 | positive |
| 50.f.Enterococcaceae | 49.f.Staphylococcaceae.g.Staphylococcus | 0.165573 | positive |
| 62.f.Enterobacteriaceae | 73.f.Staphylococcaceae.g.Staphylococcaceae | -0.16389 | negative |
| 23.o.Lactobacillales | 86.f.Lachnospiraceae | -0.1605 | negative |
| 77.f.Lachnospiraceae | 69.f.Lachnospiraceae | 0.160446 | positive |
| 42.f.Peptococcaceae.g.uncultured | 75.f.Lachnospiraceae.g.Lachnospiraceae_NK4A136_group | 0.15952 | positive |
| 19.f.Lactobacillaceae.g.HT002 | 33.f.Akkermansiaceae.g.Akkermansia | -0.15836 | negative |
| 60.f.Lachnospiraceae.g.Lachnospiraceae_UCG-001 | 59.f.Lachnospiraceae | 0.155823 | positive |
| 24.f.Clostridiaceae.g.Clostridium_sensu_stricto_1 | 51.f.Lachnospiraceae.g.Lachnospiraceae_NK4A136_group | 0.152909 | positive |
| 3.f.Lachnospiraceae.g.Roseburia | 51.f.Lachnospiraceae.g.Lachnospiraceae_NK4A136_group | -0.15238 | negative |
| 61.f.Enterobacteriaceae | 23.o.Lactobacillales | 0.152081 | positive |
| 42.f.Peptococcaceae.g.uncultured | 47.f.Lachnospiraceae.g.Lachnospiraceae_NK4A136_group | 0.150903 | positive |
| 11.f.Bacteroidaceae.g.Bacteroides | 53.f.Muribaculaceae.g.Muribaculaceae | 0.149735 | positive |
| 66.f.Bacteroidaceae.g.Bacteroides | 53.f.Muribaculaceae.g.Muribaculaceae | 0.145208 | positive |
| 49.f.Staphylococcaceae.g.Staphylococcus | 74.f.Lachnospiraceae | -0.14519 | negative |
| 42.f.Peptococcaceae.g.uncultured | 22.f.Lactobacillaceae.g.HT002 | 0.143349 | positive |
| 50.f.Enterococcaceae | 7.f.Erysipelotrichaceae.g.Turicibacter | -0.14094 | negative |
| 7.f.Erysipelotrichaceae.g.Turicibacter | 59.f.Lachnospiraceae | -0.14041 | negative |
| 24.f.Clostridiaceae.g.Clostridium_sensu_stricto_1 | 5.f.Lachnospiraceae.g.Lachnospiraceae_NK4A136_group | 0.140179 | positive |
| 7.f.Erysipelotrichaceae.g.Turicibacter | 18.f.Erysipelotrichaceae.g.Turicibacter | 0.137213 | positive |
| 66.f.Bacteroidaceae.g.Bacteroides | 55.f.Muribaculaceae.g.Muribaculaceae | 0.137191 | positive |
| 10.f.Enterobacteriaceae | 24.f.Clostridiaceae.g.Clostridium_sensu_stricto_1 | 0.136784 | positive |
| 10.f.Enterobacteriaceae | 86.f.Lachnospiraceae | 0.134459 | positive |
| 79.f.Propionibacteriaceae.g.Cutibacterium | 22.f.Lactobacillaceae.g.HT002 | 0.134333 | positive |
| 42.f.Peptococcaceae.g.uncultured | 46.f.Lachnospiraceae.g.Lachnospiraceae_NK4A136_group | 0.134181 | positive |
| 79.f.Propionibacteriaceae.g.Cutibacterium | 70.f.Clostridiaceae.g.Clostridium_sensu_stricto_1 | 0.13359 | positive |
| 38.f.Lachnospiraceae.g.Lachnospiraceae_UCG-001 | 72.f.Lachnospiraceae.g.Lachnospiraceae_NK4A136_group | -0.13347 | negative |
| 70.f.Clostridiaceae.g.Clostridium_sensu_stricto_1 | 22.f.Lactobacillaceae.g.HT002 | -0.13327 | negative |
| 24.f.Clostridiaceae.g.Clostridium_sensu_stricto_1 | 50.f.Enterococcaceae | 0.132737 | positive |
| 28.f.Enterobacteriaceae | 74.f.Lachnospiraceae | -0.13192 | negative |
| 22.f.Lactobacillaceae.g.HT002 | 6.f.Lachnospiraceae.g.Lachnospiraceae_UCG-001 | -0.13138 | negative |
| 24.f.Clostridiaceae.g.Clostridium_sensu_stricto_1 | 6.f.Lachnospiraceae.g.Lachnospiraceae_UCG-001 | 0.129211 | positive |
| 54.f.Bacteroidaceae.g.Bacteroides | 35.f.Muribaculaceae.g.Muribaculaceae | 0.128587 | positive |
| 28.f.Enterobacteriaceae | 51.f.Lachnospiraceae.g.Lachnospiraceae_NK4A136_group | -0.12701 | negative |
| 8.f.Lactobacillaceae.g.HT002 | 72.f.Lachnospiraceae.g.Lachnospiraceae_NK4A136_group | 0.126974 | positive |
| 80.f.Lachnospiraceae.g.A2 | 77.f.Lachnospiraceae | 0.124508 | positive |
| 23.o.Lactobacillales | 18.f.Erysipelotrichaceae.g.Turicibacter | 0.12425 | positive |
| 66.f.Bacteroidaceae.g.Bacteroides | 65.f.Muribaculaceae.g.Muribaculaceae | 0.122711 | positive |
| 5.f.Lachnospiraceae.g.Lachnospiraceae_NK4A136_group | 72.f.Lachnospiraceae.g.Lachnospiraceae_NK4A136_group | 0.120795 | positive |
| 61.f.Enterobacteriaceae | 24.f.Clostridiaceae.g.Clostridium_sensu_stricto_1 | 0.120261 | positive |
| 70.f.Clostridiaceae.g.Clostridium_sensu_stricto_1 | 59.f.Lachnospiraceae | 0.118437 | positive |
| 36.f.Butyricicoccaceae.g.Butyricicoccus | 77.f.Lachnospiraceae | -0.11733 | negative |
| 32.f.Monoglobaceae.g.Monoglobus | 51.f.Lachnospiraceae.g.Lachnospiraceae_NK4A136_group | -0.11606 | negative |
| 66.f.Bacteroidaceae.g.Bacteroides | 35.f.Muribaculaceae.g.Muribaculaceae | 0.115695 | positive |
| 8.f.Lactobacillaceae.g.HT002 | 33.f.Akkermansiaceae.g.Akkermansia | -0.11551 | negative |
| 23.o.Lactobacillales | 72.f.Lachnospiraceae.g.Lachnospiraceae_NK4A136_group | -0.11481 | negative |
| 28.f.Enterobacteriaceae | 59.f.Lachnospiraceae | 0.114078 | positive |
| 43.f.Clostridiaceae.g.Clostridium_sensu_stricto_1 | 13.f.Lachnospiraceae | -0.11343 | negative |
| 70.f.Clostridiaceae.g.Clostridium_sensu_stricto_1 | 47.f.Lachnospiraceae.g.Lachnospiraceae_NK4A136_group | -0.11327 | negative |
| 19.f.Lactobacillaceae.g.HT002 | 26.f.Akkermansiaceae.g.Akkermansia | -0.11273 | negative |
| 78.f.Paenibacillaceae.g.Paenibacillus | 23.o.Lactobacillales | -0.11064 | negative |
| 53.f.Muribaculaceae.g.Muribaculaceae | 40.f.Muribaculaceae.g.Muribaculaceae | -0.11035 | negative |
| 68.f.Lachnospiraceae.g.Lachnospiraceae_UCG-001 | 59.f.Lachnospiraceae | 0.10943 | positive |
| 54.f.Bacteroidaceae.g.Bacteroides | 73.f.Staphylococcaceae.g.Staphylococcaceae | -0.10908 | negative |
| 23.o.Lactobacillales | 22.f.Lactobacillaceae.g.HT002 | -0.10796 | negative |
| 70.f.Clostridiaceae.g.Clostridium_sensu_stricto_1 | 19.f.Lactobacillaceae.g.HT002 | -0.1075 | negative |
| 11.f.Bacteroidaceae.g.Bacteroides | 35.f.Muribaculaceae.g.Muribaculaceae | 0.107438 | positive |
| 61.f.Enterobacteriaceae | 73.f.Staphylococcaceae.g.Staphylococcaceae | 0.107035 | positive |
| 73.f.Staphylococcaceae.g.Staphylococcaceae | 6.f.Lachnospiraceae.g.Lachnospiraceae_UCG-001 | -0.10682 | negative |
| 11.f.Bacteroidaceae.g.Bacteroides | 65.f.Muribaculaceae.g.Muribaculaceae | 0.105349 | positive |
| 28.f.Enterobacteriaceae | 38.f.Lachnospiraceae.g.Lachnospiraceae_UCG-001 | 0.105082 | positive |
| 79.f.Propionibacteriaceae.g.Cutibacterium | 46.f.Lachnospiraceae.g.Lachnospiraceae_NK4A136_group | -0.10463 | negative |
| 28.f.Enterobacteriaceae | 68.f.Lachnospiraceae.g.Lachnospiraceae_UCG-001 | 0.104166 | positive |
| 28.f.Enterobacteriaceae | 60.f.Lachnospiraceae.g.Lachnospiraceae_UCG-001 | 0.103763 | positive |
| 43.f.Clostridiaceae.g.Clostridium_sensu_stricto_1 | 7.f.Erysipelotrichaceae.g.Turicibacter | -0.09839 | negative |
| 10.f.Enterobacteriaceae | 3.f.Lachnospiraceae.g.Roseburia | -0.09643 | negative |
| 18.f.Erysipelotrichaceae.g.Turicibacter | 74.f.Lachnospiraceae | -0.09639 | negative |
| 28.f.Enterobacteriaceae | 33.f.Akkermansiaceae.g.Akkermansia | -0.09355 | negative |
| 73.f.Staphylococcaceae.g.Staphylococcaceae | 51.f.Lachnospiraceae.g.Lachnospiraceae_NK4A136_group | -0.09349 | negative |
| 19.f.Lactobacillaceae.g.HT002 | 41.f.Akkermansiaceae.g.Akkermansia | -0.09128 | negative |
| 72.f.Lachnospiraceae.g.Lachnospiraceae_NK4A136_group | 69.f.Lachnospiraceae | 0.091187 | positive |
| 38.f.Lachnospiraceae.g.Lachnospiraceae_UCG-001 | 6.f.Lachnospiraceae.g.Lachnospiraceae_UCG-001 | 0.091094 | positive |
| 13.f.Lachnospiraceae | 77.f.Lachnospiraceae | 0.090467 | positive |
| 43.f.Clostridiaceae.g.Clostridium_sensu_stricto_1 | 80.f.Lachnospiraceae.g.A2 | -0.09007 | negative |
| 73.f.Staphylococcaceae.g.Staphylococcaceae | 5.f.Lachnospiraceae.g.Lachnospiraceae_NK4A136_group | -0.08912 | negative |
| 24.f.Clostridiaceae.g.Clostridium_sensu_stricto_1 | 64.f.Erysipelotrichaceae.g.Turicibacter | 0.086911 | positive |
| 33.f.Akkermansiaceae.g.Akkermansia | 5.f.Lachnospiraceae.g.Lachnospiraceae_NK4A136_group | -0.08676 | negative |
| 70.f.Clostridiaceae.g.Clostridium_sensu_stricto_1 | 75.f.Lachnospiraceae.g.Lachnospiraceae_NK4A136_group | -0.08675 | negative |
| 10.f.Enterobacteriaceae | 42.f.Peptococcaceae.g.uncultured | -0.0855 | negative |
| 49.f.Staphylococcaceae.g.Staphylococcus | 5.f.Lachnospiraceae.g.Lachnospiraceae_NK4A136_group | -0.08502 | negative |
| 80.f.Lachnospiraceae.g.A2 | 29.f.Lachnospiraceae | 0.083963 | positive |
| 55.f.Muribaculaceae.g.Muribaculaceae | 61.f.Enterobacteriaceae | -0.08375 | negative |
| 23.o.Lactobacillales | 7.f.Erysipelotrichaceae.g.Turicibacter | 0.083664 | positive |
| 5.f.Lachnospiraceae.g.Lachnospiraceae_NK4A136_group | 58.f.Lachnospiraceae | 0.080581 | positive |
| 53.f.Muribaculaceae.g.Muribaculaceae | 64.f.Erysipelotrichaceae.g.Turicibacter | -0.07916 | negative |
| 8.f.Lactobacillaceae.g.HT002 | 38.f.Lachnospiraceae.g.Lachnospiraceae_UCG-001 | -0.07911 | negative |
| 44.f.Muribaculaceae.g.Muribaculaceae | 24.f.Clostridiaceae.g.Clostridium_sensu_stricto_1 | -0.07884 | negative |
| 28.f.Enterobacteriaceae | 23.o.Lactobacillales | -0.07845 | negative |
| 23.o.Lactobacillales | 49.f.Staphylococcaceae.g.Staphylococcus | -0.07806 | negative |
| 36.f.Butyricicoccaceae.g.Butyricicoccus | 58.f.Lachnospiraceae | -0.0771 | negative |
| 72.f.Lachnospiraceae.g.Lachnospiraceae_NK4A136_group | 27.f.Lachnospiraceae | 0.077096 | positive |
| 50.f.Enterococcaceae | 33.f.Akkermansiaceae.g.Akkermansia | 0.077032 | positive |
| 80.f.Lachnospiraceae.g.A2 | 5.f.Lachnospiraceae.g.Lachnospiraceae_NK4A136_group | -0.07636 | negative |
| 54.f.Bacteroidaceae.g.Bacteroides | 78.f.Paenibacillaceae.g.Paenibacillus | -0.07613 | negative |
| 6.f.Lachnospiraceae.g.Lachnospiraceae_UCG-001 | 74.f.Lachnospiraceae | -0.07394 | negative |
| 22.f.Lactobacillaceae.g.HT002 | 46.f.Lachnospiraceae.g.Lachnospiraceae_NK4A136_group | 0.073837 | positive |
| 79.f.Propionibacteriaceae.g.Cutibacterium | 28.f.Enterobacteriaceae | -0.07321 | negative |
| 64.f.Erysipelotrichaceae.g.Turicibacter | 86.f.Lachnospiraceae | -0.07217 | negative |
| 61.f.Enterobacteriaceae | 18.f.Erysipelotrichaceae.g.Turicibacter | -0.07116 | negative |
| 78.f.Paenibacillaceae.g.Paenibacillus | 6.f.Lachnospiraceae.g.Lachnospiraceae_UCG-001 | -0.07101 | negative |
| 19.f.Lactobacillaceae.g.HT002 | 18.f.Erysipelotrichaceae.g.Turicibacter | 0.070221 | positive |
| 35.f.Muribaculaceae.g.Muribaculaceae | 24.f.Clostridiaceae.g.Clostridium_sensu_stricto_1 | -0.06929 | negative |
| 77.f.Lachnospiraceae | 51.f.Lachnospiraceae.g.Lachnospiraceae_NK4A136_group | 0.065942 | positive |
| 55.f.Muribaculaceae.g.Muribaculaceae | 50.f.Enterococcaceae | 0.065474 | positive |
| 7.f.Erysipelotrichaceae.g.Turicibacter | 33.f.Akkermansiaceae.g.Akkermansia | -0.06454 | negative |
| 22.f.Lactobacillaceae.g.HT002 | 73.f.Staphylococcaceae.g.Staphylococcaceae | 0.064434 | positive |
| 7.f.Erysipelotrichaceae.g.Turicibacter | 38.f.Lachnospiraceae.g.Lachnospiraceae_UCG-001 | -0.0644 | negative |
| 53.f.Muribaculaceae.g.Muribaculaceae | 46.f.Lachnospiraceae.g.Lachnospiraceae_NK4A136_group | -0.06429 | negative |
| 23.o.Lactobacillales | 51.f.Lachnospiraceae.g.Lachnospiraceae_NK4A136_group | -0.0641 | negative |
| 23.o.Lactobacillales | 77.f.Lachnospiraceae | -0.06379 | negative |
| 70.f.Clostridiaceae.g.Clostridium_sensu_stricto_1 | 5.f.Lachnospiraceae.g.Lachnospiraceae_NK4A136_group | 0.062558 | positive |
| 40.f.Muribaculaceae.g.Muribaculaceae | 19.f.Lactobacillaceae.g.HT002 | 0.062345 | positive |
| 22.f.Lactobacillaceae.g.HT002 | 77.f.Lachnospiraceae | -0.0623 | negative |
| 6.f.Lachnospiraceae.g.Lachnospiraceae_UCG-001 | 59.f.Lachnospiraceae | 0.062294 | positive |
| 46.f.Lachnospiraceae.g.Lachnospiraceae_NK4A136_group | 58.f.Lachnospiraceae | 0.062237 | positive |
| 60.f.Lachnospiraceae.g.Lachnospiraceae_UCG-001 | 6.f.Lachnospiraceae.g.Lachnospiraceae_UCG-001 | 0.061731 | positive |
| 28.f.Enterobacteriaceae | 49.f.Staphylococcaceae.g.Staphylococcus | 0.061604 | positive |
| 42.f.Peptococcaceae.g.uncultured | 64.f.Erysipelotrichaceae.g.Turicibacter | -0.06003 | negative |
| 36.f.Butyricicoccaceae.g.Butyricicoccus | 27.f.Lachnospiraceae | -0.05979 | negative |
| 8.f.Lactobacillaceae.g.HT002 | 7.f.Erysipelotrichaceae.g.Turicibacter | 0.059542 | positive |
| 68.f.Lachnospiraceae.g.Lachnospiraceae_UCG-001 | 80.f.Lachnospiraceae.g.A2 | 0.059081 | positive |
| 33.f.Akkermansiaceae.g.Akkermansia | 68.f.Lachnospiraceae.g.Lachnospiraceae_UCG-001 | 0.059046 | positive |
| 78.f.Paenibacillaceae.g.Paenibacillus | 38.f.Lachnospiraceae.g.Lachnospiraceae_UCG-001 | -0.05874 | negative |
| 79.f.Propionibacteriaceae.g.Cutibacterium | 49.f.Staphylococcaceae.g.Staphylococcus | 0.058445 | positive |
| 43.f.Clostridiaceae.g.Clostridium_sensu_stricto_1 | 32.f.Monoglobaceae.g.Monoglobus | -0.05832 | negative |
| 53.f.Muribaculaceae.g.Muribaculaceae | 38.f.Lachnospiraceae.g.Lachnospiraceae_UCG-001 | -0.05815 | negative |
| 65.f.Muribaculaceae.g.Muribaculaceae | 50.f.Enterococcaceae | -0.05809 | negative |
| 78.f.Paenibacillaceae.g.Paenibacillus | 22.f.Lactobacillaceae.g.HT002 | 0.057601 | positive |
| 49.f.Staphylococcaceae.g.Staphylococcus | 75.f.Lachnospiraceae.g.Lachnospiraceae_NK4A136_group | -0.05752 | negative |
| 53.f.Muribaculaceae.g.Muribaculaceae | 6.f.Lachnospiraceae.g.Lachnospiraceae_UCG-001 | -0.05736 | negative |
| 40.f.Muribaculaceae.g.Muribaculaceae | 86.f.Lachnospiraceae | 0.056991 | positive |
| 32.f.Monoglobaceae.g.Monoglobus | 77.f.Lachnospiraceae | 0.056779 | positive |
| 10.f.Enterobacteriaceae | 72.f.Lachnospiraceae.g.Lachnospiraceae_NK4A136_group | -0.05654 | negative |
| 10.f.Enterobacteriaceae | 73.f.Staphylococcaceae.g.Staphylococcaceae | 0.056291 | positive |
| 24.f.Clostridiaceae.g.Clostridium_sensu_stricto_1 | 7.f.Erysipelotrichaceae.g.Turicibacter | -0.05627 | negative |
| 33.f.Akkermansiaceae.g.Akkermansia | 59.f.Lachnospiraceae | 0.055475 | positive |
| 78.f.Paenibacillaceae.g.Paenibacillus | 49.f.Staphylococcaceae.g.Staphylococcus | 0.055333 | positive |
| 54.f.Bacteroidaceae.g.Bacteroides | 70.f.Clostridiaceae.g.Clostridium_sensu_stricto_1 | -0.05519 | negative |
| 51.f.Lachnospiraceae.g.Lachnospiraceae_NK4A136_group | 29.f.Lachnospiraceae | -0.05516 | negative |
| 18.f.Erysipelotrichaceae.g.Turicibacter | 86.f.Lachnospiraceae | -0.05488 | negative |
| 53.f.Muribaculaceae.g.Muribaculaceae | 24.f.Clostridiaceae.g.Clostridium_sensu_stricto_1 | -0.05398 | negative |
| 10.f.Enterobacteriaceae | 49.f.Staphylococcaceae.g.Staphylococcus | 0.053898 | positive |
| 49.f.Staphylococcaceae.g.Staphylococcus | 47.f.Lachnospiraceae.g.Lachnospiraceae_NK4A136_group | -0.05367 | negative |
| 19.f.Lactobacillaceae.g.HT002 | 73.f.Staphylococcaceae.g.Staphylococcaceae | -0.05347 | negative |
| 47.f.Lachnospiraceae.g.Lachnospiraceae_NK4A136_group | 58.f.Lachnospiraceae | 0.053165 | positive |
| 10.f.Enterobacteriaceae | 29.f.Lachnospiraceae | -0.05286 | negative |
| 53.f.Muribaculaceae.g.Muribaculaceae | 28.f.Enterobacteriaceae | -0.05269 | negative |
| 74.f.Lachnospiraceae | 59.f.Lachnospiraceae | -0.05258 | negative |
| 10.f.Enterobacteriaceae | 18.f.Erysipelotrichaceae.g.Turicibacter | -0.0521 | negative |
| 61.f.Enterobacteriaceae | 38.f.Lachnospiraceae.g.Lachnospiraceae_UCG-001 | -0.05183 | negative |
| 65.f.Muribaculaceae.g.Muribaculaceae | 55.f.Muribaculaceae.g.Muribaculaceae | 0.051573 | positive |
| 22.f.Lactobacillaceae.g.HT002 | 47.f.Lachnospiraceae.g.Lachnospiraceae_NK4A136_group | 0.050956 | positive |
| 77.f.Lachnospiraceae | 29.f.Lachnospiraceae | 0.050854 | positive |
| UNTREATED HOM MICE | | | |
| 743.f.Muribaculaceae.g.Muribaculaceae | 168.f.Muribaculaceae.g.Muribaculaceae | 0.092195 | positive |
| 743.f.Muribaculaceae.g.Muribaculaceae | 222.f.Clostridia_UCG-014.g.Clostridia_UCG-014 | 0.253912 | positive |
| 964.f.Muribaculaceae.g.Muribaculaceae | 241.f.Lachnospiraceae.g.Lachnospiraceae_NK4A136_group | -0.20938 | negative |
| 577.f.Muribaculaceae.g.Muribaculaceae | 262.f.Acholeplasmataceae.g.A.naeroplasma | -0.26856 | negative |
| 743.f.Muribaculaceae.g.Muribaculaceae | 262.f.Acholeplasmataceae.g.A.naeroplasma | -0.07197 | negative |
| 577.f.Muribaculaceae.g.Muribaculaceae | 268.f.Lachnospiraceae.na | -0.17805 | negative |
| 743.f.Muribaculaceae.g.Muribaculaceae | 268.f.Lachnospiraceae.na | -0.09165 | negative |
| 964.f.Muribaculaceae.g.Muribaculaceae | 268.f.Lachnospiraceae.na | -0.07246 | negative |
| 577.f.Muribaculaceae.g.Muribaculaceae | 309.f.Lachnospiraceae.na | -0.23149 | negative |
| 743.f.Muribaculaceae.g.Muribaculaceae | 355.f.Lachnospiraceae.na | -0.10289 | negative |
| 964.f.Muribaculaceae.g.Muribaculaceae | 370.f.Lachnospiraceae.g.Lachnospiraceae_NK4A136_group | 0.116831 | positive |
| 577.f.Muribaculaceae.g.Muribaculaceae | 487.f.Lachnospiraceae.g.Lachnospiraceae_NK4A136_group | -0.13123 | negative |
| 743.f.Muribaculaceae.g.Muribaculaceae | 487.f.Lachnospiraceae.g.Lachnospiraceae_NK4A136_group | -0.15054 | negative |
| 743.f.Muribaculaceae.g.Muribaculaceae | 536.f.Lachnospiraceae.g.Lachnospiraceae_NK4A136_group | -0.14214 | negative |
| 577.f.Muribaculaceae.g.Muribaculaceae | 574.f.Bacteroidaceae.g.Bacteroides | 0.117579 | positive |
| 743.f.Muribaculaceae.g.Muribaculaceae | 574.f.Bacteroidaceae.g.Bacteroides | 0.129226 | positive |
| 743.f.Muribaculaceae.g.Muribaculaceae | 577.f.Muribaculaceae.g.Muribaculaceae | 0.082943 | positive |
| 964.f.Muribaculaceae.g.Muribaculaceae | 610.f.Ruminococcaceae.g.Ruminococcus | -0.13497 | negative |
| 577.f.Muribaculaceae.g.Muribaculaceae | 616.f.Muribaculaceae.g.Muribaculaceae | 0.084978 | positive |
| 743.f.Muribaculaceae.g.Muribaculaceae | 616.f.Muribaculaceae.g.Muribaculaceae | 0.1087 | positive |
| 743.f.Muribaculaceae.g.Muribaculaceae | 964.f.Muribaculaceae.g.Muribaculaceae | 0.078118 | positive |
| 358.f.Akkermansiaceae.g.Akkermansia | 577.f.Muribaculaceae.g.Muribaculaceae | 0.18988 | positive |
| 815.f.Muribaculaceae.g.Muribaculaceae | 577.f.Muribaculaceae.g.Muribaculaceae | 0.078552 | positive |
| 168.f.Muribaculaceae.g.Muribaculaceae | 964.f.Muribaculaceae.g.Muribaculaceae | 0.111969 | positive |
| 574.f.Bacteroidaceae.g.Bacteroides | 964.f.Muribaculaceae.g.Muribaculaceae | 0.224145 | positive |
| 616.f.Muribaculaceae.g.Muribaculaceae | 964.f.Muribaculaceae.g.Muribaculaceae | 0.091488 | positive |
| 168.f.Muribaculaceae.g.Muribaculaceae | 222.f.Clostridia_UCG-014.g.Clostridia_UCG-014 | 0.075018 | positive |
| 170.f.Rikenellaceae.g.Alistipes | 222.f.Clostridia_UCG-014.g.Clostridia_UCG-014 | -0.06979 | negative |
| 268.f.Lachnospiraceae.na | 222.f.Clostridia_UCG-014.g.Clostridia_UCG-014 | 0.382291 | positive |
| 355.f.Lachnospiraceae.na | 222.f.Clostridia_UCG-014.g.Clostridia_UCG-014 | -0.10307 | negative |
| 358.f.Akkermansiaceae.g.Akkermansia | 222.f.Clostridia_UCG-014.g.Clostridia_UCG-014 | -0.24913 | negative |
| 663.f.Erysipelotrichaceae.g.Turicibacter | 222.f.Clostridia_UCG-014.g.Clostridia_UCG-014 | -0.10701 | negative |
| 815.f.Muribaculaceae.g.Muribaculaceae | 222.f.Clostridia_UCG-014.g.Clostridia_UCG-014 | 0.178199 | positive |
| 894.f.Clostridia_vadinBB60_group.g.Clostridia_vadinBB60_group | 222.f.Clostridia_UCG-014.g.Clostridia_UCG-014 | -0.10139 | negative |
| 268.f.Lachnospiraceae.na | 241.f.Lachnospiraceae.g.Lachnospiraceae_NK4A136_group | 0.072212 | positive |
| 355.f.Lachnospiraceae.na | 241.f.Lachnospiraceae.g.Lachnospiraceae_NK4A136_group | -0.09668 | negative |
| 358.f.Akkermansiaceae.g.Akkermansia | 241.f.Lachnospiraceae.g.Lachnospiraceae_NK4A136_group | -0.14762 | negative |
| 46.f.Lachnospiraceae.g.Lachnospiraceae_NK4A136_group | 241.f.Lachnospiraceae.g.Lachnospiraceae_NK4A136_group | 0.118116 | positive |
| 894.f.Clostridia_vadinBB60_group.g.Clostridia_vadinBB60_group | 241.f.Lachnospiraceae.g.Lachnospiraceae_NK4A136_group | -0.22359 | negative |
| 940.f.Lachnospiraceae.na | 241.f.Lachnospiraceae.g.Lachnospiraceae_NK4A136_group | -0.20707 | negative |
| 944.f.Muribaculaceae.g.Muribaculaceae | 241.f.Lachnospiraceae.g.Lachnospiraceae_NK4A136_group | -0.07066 | negative |
| 172.f.Sutterellaceae.g.Parasutterella | 262.f.Acholeplasmataceae.g.A.naeroplasma | -0.19137 | negative |
| 222.f.Clostridia_UCG-014.g.Clostridia_UCG-014 | 262.f.Acholeplasmataceae.g.A.naeroplasma | 0.089114 | positive |
| 241.f.Lachnospiraceae.g.Lachnospiraceae_NK4A136_group | 262.f.Acholeplasmataceae.g.A.naeroplasma | -0.16613 | negative |
| 271.f.Lactobacillaceae.na | 262.f.Acholeplasmataceae.g.A.naeroplasma | -0.1643 | negative |
| 355.f.Lachnospiraceae.na | 262.f.Acholeplasmataceae.g.A.naeroplasma | 0.374696 | positive |
| 358.f.Akkermansiaceae.g.Akkermansia | 262.f.Acholeplasmataceae.g.A.naeroplasma | 0.068766 | positive |
| 46.f.Lachnospiraceae.g.Lachnospiraceae_NK4A136_group | 262.f.Acholeplasmataceae.g.A.naeroplasma | -0.07615 | negative |
| 536.f.Lachnospiraceae.g.Lachnospiraceae_NK4A136_group | 262.f.Acholeplasmataceae.g.A.naeroplasma | -0.16851 | negative |
| 616.f.Muribaculaceae.g.Muribaculaceae | 262.f.Acholeplasmataceae.g.A.naeroplasma | -0.14139 | negative |
| 663.f.Erysipelotrichaceae.g.Turicibacter | 262.f.Acholeplasmataceae.g.A.naeroplasma | -0.11677 | negative |
| 894.f.Clostridia_vadinBB60_group.g.Clostridia_vadinBB60_group | 262.f.Acholeplasmataceae.g.A.naeroplasma | 0.081035 | positive |
| 940.f.Lachnospiraceae.na | 262.f.Acholeplasmataceae.g.A.naeroplasma | -0.10749 | negative |
| 944.f.Muribaculaceae.g.Muribaculaceae | 262.f.Acholeplasmataceae.g.A.naeroplasma | -0.24153 | negative |
| 355.f.Lachnospiraceae.na | 268.f.Lachnospiraceae.na | 0.097814 | positive |
| 358.f.Akkermansiaceae.g.Akkermansia | 268.f.Lachnospiraceae.na | -0.07355 | negative |
| 536.f.Lachnospiraceae.g.Lachnospiraceae_NK4A136_group | 268.f.Lachnospiraceae.na | 0.112447 | positive |
| 574.f.Bacteroidaceae.g.Bacteroides | 268.f.Lachnospiraceae.na | -0.15363 | negative |
| 894.f.Clostridia_vadinBB60_group.g.Clostridia_vadinBB60_group | 268.f.Lachnospiraceae.na | -0.29147 | negative |
| 940.f.Lachnospiraceae.na | 268.f.Lachnospiraceae.na | 0.095582 | positive |
| 46.f.Lachnospiraceae.g.Lachnospiraceae_NK4A136_group | 271.f.Lactobacillaceae.na | -0.08443 | negative |
| 574.f.Bacteroidaceae.g.Bacteroides | 271.f.Lactobacillaceae.na | -0.06757 | negative |
| 815.f.Muribaculaceae.g.Muribaculaceae | 271.f.Lactobacillaceae.na | 0.317571 | positive |
| 894.f.Clostridia_vadinBB60_group.g.Clostridia_vadinBB60_group | 271.f.Lactobacillaceae.na | -0.15692 | negative |
| 170.f.Rikenellaceae.g.Alistipes | 309.f.Lachnospiraceae.na | 0.122778 | positive |
| 172.f.Sutterellaceae.g.Parasutterella | 309.f.Lachnospiraceae.na | 0.060427 | positive |
| 241.f.Lachnospiraceae.g.Lachnospiraceae_NK4A136_group | 309.f.Lachnospiraceae.na | 0.175035 | positive |
| 268.f.Lachnospiraceae.na | 309.f.Lachnospiraceae.na | -0.07616 | negative |
| 355.f.Lachnospiraceae.na | 309.f.Lachnospiraceae.na | -0.43677 | negative |
| 358.f.Akkermansiaceae.g.Akkermansia | 309.f.Lachnospiraceae.na | -0.06644 | negative |
| 536.f.Lachnospiraceae.g.Lachnospiraceae_NK4A136_group | 309.f.Lachnospiraceae.na | -0.25894 | negative |
| 610.f.Ruminococcaceae.g.Ruminococcus | 309.f.Lachnospiraceae.na | -0.09759 | negative |
| 663.f.Erysipelotrichaceae.g.Turicibacter | 309.f.Lachnospiraceae.na | 0.246643 | positive |
| 675.f.Muribaculaceae.g.Muribaculaceae | 309.f.Lachnospiraceae.na | -0.09605 | negative |
| 750.f.Muribaculaceae.g.Muribaculaceae | 309.f.Lachnospiraceae.na | -0.10028 | negative |
| 894.f.Clostridia_vadinBB60_group.g.Clostridia_vadinBB60_group | 309.f.Lachnospiraceae.na | -0.09813 | negative |
| 944.f.Muribaculaceae.g.Muribaculaceae | 309.f.Lachnospiraceae.na | 0.182867 | positive |
| 271.f.Lactobacillaceae.na | 355.f.Lachnospiraceae.na | 0.105108 | positive |
| 358.f.Akkermansiaceae.g.Akkermansia | 355.f.Lachnospiraceae.na | -0.06789 | negative |
| 574.f.Bacteroidaceae.g.Bacteroides | 355.f.Lachnospiraceae.na | -0.08016 | negative |
| 675.f.Muribaculaceae.g.Muribaculaceae | 355.f.Lachnospiraceae.na | -0.07063 | negative |
| 815.f.Muribaculaceae.g.Muribaculaceae | 355.f.Lachnospiraceae.na | 0.078405 | positive |
| 894.f.Clostridia_vadinBB60_group.g.Clostridia_vadinBB60_group | 355.f.Lachnospiraceae.na | 0.060388 | positive |
| 944.f.Muribaculaceae.g.Muribaculaceae | 358.f.Akkermansiaceae.g.Akkermansia | 0.121273 | positive |
| 222.f.Clostridia_UCG-014.g.Clostridia_UCG-014 | 370.f.Lachnospiraceae.g.Lachnospiraceae_NK4A136_group | -0.31875 | negative |
| 262.f.Acholeplasmataceae.g.A.naeroplasma | 370.f.Lachnospiraceae.g.Lachnospiraceae_NK4A136_group | 0.193361 | positive |
| 268.f.Lachnospiraceae.na | 370.f.Lachnospiraceae.g.Lachnospiraceae_NK4A136_group | -0.16026 | negative |
| 271.f.Lactobacillaceae.na | 370.f.Lachnospiraceae.g.Lachnospiraceae_NK4A136_group | -0.16909 | negative |
| 309.f.Lachnospiraceae.na | 370.f.Lachnospiraceae.g.Lachnospiraceae_NK4A136_group | 0.120393 | positive |
| 355.f.Lachnospiraceae.na | 370.f.Lachnospiraceae.g.Lachnospiraceae_NK4A136_group | -0.18814 | negative |
| 610.f.Ruminococcaceae.g.Ruminococcus | 370.f.Lachnospiraceae.g.Lachnospiraceae_NK4A136_group | 0.124912 | positive |
| 663.f.Erysipelotrichaceae.g.Turicibacter | 370.f.Lachnospiraceae.g.Lachnospiraceae_NK4A136_group | -0.16117 | negative |
| 675.f.Muribaculaceae.g.Muribaculaceae | 370.f.Lachnospiraceae.g.Lachnospiraceae_NK4A136_group | -0.09037 | negative |
| 742.f.RF39.g.RF39 | 370.f.Lachnospiraceae.g.Lachnospiraceae_NK4A136_group | -0.05499 | negative |
| 750.f.Muribaculaceae.g.Muribaculaceae | 370.f.Lachnospiraceae.g.Lachnospiraceae_NK4A136_group | -0.07943 | negative |
| 815.f.Muribaculaceae.g.Muribaculaceae | 370.f.Lachnospiraceae.g.Lachnospiraceae_NK4A136_group | -0.056 | negative |
| 894.f.Clostridia_vadinBB60_group.g.Clostridia_vadinBB60_group | 370.f.Lachnospiraceae.g.Lachnospiraceae_NK4A136_group | -0.22884 | negative |
| 944.f.Muribaculaceae.g.Muribaculaceae | 370.f.Lachnospiraceae.g.Lachnospiraceae_NK4A136_group | 0.094165 | positive |
| 241.f.Lachnospiraceae.g.Lachnospiraceae_NK4A136_group | 408.f.Lachnospiraceae.g.Lachnospiraceae_NK4A136_group | 0.111979 | positive |
| 46.f.Lachnospiraceae.g.Lachnospiraceae_NK4A136_group | 408.f.Lachnospiraceae.g.Lachnospiraceae_NK4A136_group | 0.639228 | positive |
| 536.f.Lachnospiraceae.g.Lachnospiraceae_NK4A136_group | 408.f.Lachnospiraceae.g.Lachnospiraceae_NK4A136_group | 0.193544 | positive |
| 894.f.Clostridia_vadinBB60_group.g.Clostridia_vadinBB60_group | 408.f.Lachnospiraceae.g.Lachnospiraceae_NK4A136_group | 0.103574 | positive |
| 168.f.Muribaculaceae.g.Muribaculaceae | 46.f.Lachnospiraceae.g.Lachnospiraceae_NK4A136_group | -0.05903 | negative |
| 536.f.Lachnospiraceae.g.Lachnospiraceae_NK4A136_group | 46.f.Lachnospiraceae.g.Lachnospiraceae_NK4A136_group | 0.299686 | positive |
| 616.f.Muribaculaceae.g.Muribaculaceae | 46.f.Lachnospiraceae.g.Lachnospiraceae_NK4A136_group | -0.05564 | negative |
| 750.f.Muribaculaceae.g.Muribaculaceae | 46.f.Lachnospiraceae.g.Lachnospiraceae_NK4A136_group | -0.05322 | negative |
| 168.f.Muribaculaceae.g.Muribaculaceae | 487.f.Lachnospiraceae.g.Lachnospiraceae_NK4A136_group | -0.11034 | negative |
| 170.f.Rikenellaceae.g.Alistipes | 487.f.Lachnospiraceae.g.Lachnospiraceae_NK4A136_group | -0.18881 | negative |
| 172.f.Sutterellaceae.g.Parasutterella | 487.f.Lachnospiraceae.g.Lachnospiraceae_NK4A136_group | -0.23394 | negative |
| 222.f.Clostridia_UCG-014.g.Clostridia_UCG-014 | 487.f.Lachnospiraceae.g.Lachnospiraceae_NK4A136_group | -0.20588 | negative |
| 241.f.Lachnospiraceae.g.Lachnospiraceae_NK4A136_group | 487.f.Lachnospiraceae.g.Lachnospiraceae_NK4A136_group | -0.1295 | negative |
| 262.f.Acholeplasmataceae.g.A.naeroplasma | 487.f.Lachnospiraceae.g.Lachnospiraceae_NK4A136_group | -0.13497 | negative |
| 268.f.Lachnospiraceae.na | 487.f.Lachnospiraceae.g.Lachnospiraceae_NK4A136_group | -0.135 | negative |
| 271.f.Lactobacillaceae.na | 487.f.Lachnospiraceae.g.Lachnospiraceae_NK4A136_group | 0.067335 | positive |
| 309.f.Lachnospiraceae.na | 487.f.Lachnospiraceae.g.Lachnospiraceae_NK4A136_group | 0.081305 | positive |
| 358.f.Akkermansiaceae.g.Akkermansia | 487.f.Lachnospiraceae.g.Lachnospiraceae_NK4A136_group | -0.15672 | negative |
| 536.f.Lachnospiraceae.g.Lachnospiraceae_NK4A136_group | 487.f.Lachnospiraceae.g.Lachnospiraceae_NK4A136_group | 0.112454 | positive |
| 616.f.Muribaculaceae.g.Muribaculaceae | 487.f.Lachnospiraceae.g.Lachnospiraceae_NK4A136_group | -0.07544 | negative |
| 750.f.Muribaculaceae.g.Muribaculaceae | 487.f.Lachnospiraceae.g.Lachnospiraceae_NK4A136_group | 0.08144 | positive |
| 894.f.Clostridia_vadinBB60_group.g.Clostridia_vadinBB60_group | 487.f.Lachnospiraceae.g.Lachnospiraceae_NK4A136_group | -0.05977 | negative |
| 168.f.Muribaculaceae.g.Muribaculaceae | 536.f.Lachnospiraceae.g.Lachnospiraceae_NK4A136_group | -0.1781 | negative |
| 170.f.Rikenellaceae.g.Alistipes | 536.f.Lachnospiraceae.g.Lachnospiraceae_NK4A136_group | -0.1247 | negative |
| 815.f.Muribaculaceae.g.Muribaculaceae | 536.f.Lachnospiraceae.g.Lachnospiraceae_NK4A136_group | -0.08387 | negative |
| 358.f.Akkermansiaceae.g.Akkermansia | 574.f.Bacteroidaceae.g.Bacteroides | 0.299225 | positive |
| 616.f.Muribaculaceae.g.Muribaculaceae | 574.f.Bacteroidaceae.g.Bacteroides | 0.122135 | positive |
| 170.f.Rikenellaceae.g.Alistipes | 610.f.Ruminococcaceae.g.Ruminococcus | -0.32819 | negative |
| 355.f.Lachnospiraceae.na | 610.f.Ruminococcaceae.g.Ruminococcus | -0.09851 | negative |
| 358.f.Akkermansiaceae.g.Akkermansia | 610.f.Ruminococcaceae.g.Ruminococcus | 0.05936 | positive |
| 815.f.Muribaculaceae.g.Muribaculaceae | 610.f.Ruminococcaceae.g.Ruminococcus | 0.06554 | positive |
| 894.f.Clostridia_vadinBB60_group.g.Clostridia_vadinBB60_group | 610.f.Ruminococcaceae.g.Ruminococcus | -0.32317 | negative |
| 168.f.Muribaculaceae.g.Muribaculaceae | 616.f.Muribaculaceae.g.Muribaculaceae | 0.150621 | positive |
| 168.f.Muribaculaceae.g.Muribaculaceae | 663.f.Erysipelotrichaceae.g.Turicibacter | -0.1127 | negative |
| 170.f.Rikenellaceae.g.Alistipes | 663.f.Erysipelotrichaceae.g.Turicibacter | -0.09092 | negative |
| 172.f.Sutterellaceae.g.Parasutterella | 663.f.Erysipelotrichaceae.g.Turicibacter | 0.147913 | positive |
| 241.f.Lachnospiraceae.g.Lachnospiraceae_NK4A136_group | 663.f.Erysipelotrichaceae.g.Turicibacter | -0.44967 | negative |
| 268.f.Lachnospiraceae.na | 663.f.Erysipelotrichaceae.g.Turicibacter | 0.077242 | positive |
| 408.f.Lachnospiraceae.g.Lachnospiraceae_NK4A136_group | 663.f.Erysipelotrichaceae.g.Turicibacter | -0.05186 | negative |
| 46.f.Lachnospiraceae.g.Lachnospiraceae_NK4A136_group | 663.f.Erysipelotrichaceae.g.Turicibacter | -0.07908 | negative |
| 536.f.Lachnospiraceae.g.Lachnospiraceae_NK4A136_group | 663.f.Erysipelotrichaceae.g.Turicibacter | -0.10842 | negative |
| 574.f.Bacteroidaceae.g.Bacteroides | 663.f.Erysipelotrichaceae.g.Turicibacter | -0.13802 | negative |
| 815.f.Muribaculaceae.g.Muribaculaceae | 663.f.Erysipelotrichaceae.g.Turicibacter | 0.053694 | positive |
| 940.f.Lachnospiraceae.na | 663.f.Erysipelotrichaceae.g.Turicibacter | 0.250004 | positive |
| 536.f.Lachnospiraceae.g.Lachnospiraceae_NK4A136_group | 675.f.Muribaculaceae.g.Muribaculaceae | -0.08152 | negative |
| 815.f.Muribaculaceae.g.Muribaculaceae | 675.f.Muribaculaceae.g.Muribaculaceae | -0.14353 | negative |
| 940.f.Lachnospiraceae.na | 675.f.Muribaculaceae.g.Muribaculaceae | -0.06947 | negative |
| 944.f.Muribaculaceae.g.Muribaculaceae | 675.f.Muribaculaceae.g.Muribaculaceae | -0.06028 | negative |
| 170.f.Rikenellaceae.g.Alistipes | 742.f.RF39.g.RF39 | -0.11271 | negative |
| 222.f.Clostridia_UCG-014.g.Clostridia_UCG-014 | 742.f.RF39.g.RF39 | 0.057939 | positive |
| 241.f.Lachnospiraceae.g.Lachnospiraceae_NK4A136_group | 742.f.RF39.g.RF39 | -0.11994 | negative |
| 268.f.Lachnospiraceae.na | 742.f.RF39.g.RF39 | -0.15524 | negative |
| 309.f.Lachnospiraceae.na | 742.f.RF39.g.RF39 | -0.05992 | negative |
| 355.f.Lachnospiraceae.na | 742.f.RF39.g.RF39 | -0.19953 | negative |
| 358.f.Akkermansiaceae.g.Akkermansia | 742.f.RF39.g.RF39 | -0.24616 | negative |
| 487.f.Lachnospiraceae.g.Lachnospiraceae_NK4A136_group | 742.f.RF39.g.RF39 | 0.25936 | positive |
| 610.f.Ruminococcaceae.g.Ruminococcus | 742.f.RF39.g.RF39 | -0.13765 | negative |
| 616.f.Muribaculaceae.g.Muribaculaceae | 742.f.RF39.g.RF39 | -0.09182 | negative |
| 663.f.Erysipelotrichaceae.g.Turicibacter | 742.f.RF39.g.RF39 | 0.234807 | positive |
| 815.f.Muribaculaceae.g.Muribaculaceae | 742.f.RF39.g.RF39 | 0.068694 | positive |
| 894.f.Clostridia_vadinBB60_group.g.Clostridia_vadinBB60_group | 742.f.RF39.g.RF39 | 0.150218 | positive |
| 940.f.Lachnospiraceae.na | 742.f.RF39.g.RF39 | -0.31838 | negative |
| 944.f.Muribaculaceae.g.Muribaculaceae | 742.f.RF39.g.RF39 | -0.12613 | negative |
| 675.f.Muribaculaceae.g.Muribaculaceae | 750.f.Muribaculaceae.g.Muribaculaceae | 0.848948 | positive |
| 815.f.Muribaculaceae.g.Muribaculaceae | 750.f.Muribaculaceae.g.Muribaculaceae | -0.14655 | negative |
| 46.f.Lachnospiraceae.g.Lachnospiraceae_NK4A136_group | 894.f.Clostridia_vadinBB60_group.g.Clostridia_vadinBB60_group | 0.080638 | positive |
| 815.f.Muribaculaceae.g.Muribaculaceae | 894.f.Clostridia_vadinBB60_group.g.Clostridia_vadinBB60_group | 0.08044 | positive |
| 944.f.Muribaculaceae.g.Muribaculaceae | 894.f.Clostridia_vadinBB60_group.g.Clostridia_vadinBB60_group | -0.22168 | negative |
| 815.f.Muribaculaceae.g.Muribaculaceae | 940.f.Lachnospiraceae.na | -0.24833 | negative |

**Supplementary Table 2.** | Co-Abundance Network values showing node connections, rho (strength of connection), and negative or positive association of Hom.ABX.DM mice, *n* = 17; and untreated Hom mice *n* = 16.
