# Supplementary material for: Perinatal antibiotics in a db/db mouse model impact obesity and hyperglycemia in a microbiome-dependent manner
Source: Front Microbiomes. 2026 Jul 27;5:1750320. doi: 10.3389/frmbi.2026.1750320 (PMC13454812; doi:10.3389/frmbi.2026.1750320)
Supplement: Supplementary file 4 [file Supplementaryfile2.docx]

**Supplementary Figure S2. | A.** Female weights including WT mice. *n* = 4-16 per timepoint per genotype; WT mice *n* = 3-5 per timepoint per genotype. **B.** Female glucose including WT mice. *n* = 4-16 per timepoint per genotype; WT mice *n* = 1-5 per timepoint per genotype. **C.** Male weights including WT mice. *n* = 4-16 per timepoint per genotype; WT mice *n* = 2-10 per timepoint per genotype. **D.** Male glucose including WT mice. *n* = 4-16 per timepoint per genotype; WT mice *n* = 1-8 per timepoint per genotype. (A-D all weights for Het-, Hom-, Het+, and Hom+ are repeated from Figure 2.)
