# Supplementary material for: Perinatal antibiotics in a db/db mouse model impact obesity and hyperglycemia in a microbiome-dependent manner
Source: Front Microbiomes. 2026 Jul 27;5:1750320. doi: 10.3389/frmbi.2026.1750320 (PMC13454812; doi:10.3389/frmbi.2026.1750320)
Supplement: Supplementary file 6 [file Supplementaryfile4.docx]

**Supplementary Figure S4. |** Bray-Curtis dissimilarity of combined female and male Het versus Hom mice. Significance measured via PERMANOVA. *Significance values as follows: ns p ≥ 0.05, * p = 0.01 to 0.05, ** p = 0.001 to 0.01, *** p = 0.0001 to 0.001, **** p < 0.0001. n* = 37-83.
