# Supplementary material for: Perinatal antibiotics in a db/db mouse model impact obesity and hyperglycemia in a microbiome-dependent manner
Source: Front Microbiomes. 2026 Jul 27;5:1750320. doi: 10.3389/frmbi.2026.1750320 (PMC13454812; doi:10.3389/frmbi.2026.1750320)
Supplement: Supplementary file 7 [file Supplementaryfile5.docx]

q@

**Supplementary Figure S5. |** Shannon diversity of female and male, treated and untreated, Het and Hom mice. Significance calculated using Two-way ANOVA. Significance measured via PERMANOVA. *n* = 16-49 per group. *p* = 0.0000.
