# Supplementary material for: Perinatal antibiotics in a db/db mouse model impact obesity and hyperglycemia in a microbiome-dependent manner
Source: Front Microbiomes. 2026 Jul 27;5:1750320. doi: 10.3389/frmbi.2026.1750320 (PMC13454812; doi:10.3389/frmbi.2026.1750320)
Supplement: Supplementary file 8 [file Supplementaryfile6.docx]

**Supplementary Figure S6. | A.** Relative abundance of Het mice at 8-weeks-old. *n* = 5-11 per group. **B.** Relative abundance of Hom mice at 8-weeks-old. *n* = 4-11 per group.
