# Supplementary material for: Perinatal antibiotics in a db/db mouse model impact obesity and hyperglycemia in a microbiome-dependent manner
Source: Front Microbiomes. 2026 Jul 27;5:1750320. doi: 10.3389/frmbi.2026.1750320 (PMC13454812; doi:10.3389/frmbi.2026.1750320)
Supplement: Supplementary file 10 [file Supplementaryfile8.docx]

**Supplementary Figure S8. | A.** DESeq2 of female Hom treated versus untreated mice at 13-weeks-old. *n* = 4-5 per treatment. **B.** DESeq2 of male Hom treated versus untreated mice at 13-weeks-old. *n* = 5 per treatment.
