# Supplementary material for: Perinatal antibiotics in a db/db mouse model impact obesity and hyperglycemia in a microbiome-dependent manner
Source: Front Microbiomes. 2026 Jul 27;5:1750320. doi: 10.3389/frmbi.2026.1750320 (PMC13454812; doi:10.3389/frmbi.2026.1750320)
Supplement: Supplementary file 11 [file Supplementaryfile9.docx]

| ***D. muris* ASVs** | **5** | **8** | **10** | **13** | **5+ABX** | **8+ABX** | **10+ABX** | **13+ABX** |
| --- | --- | --- | --- | --- | --- | --- | --- | --- |
| **11.f.Muribaculaceae.g.Muribaculaceae** | 0.00% | 0.00% | 0.00% | 0.00% | 0.00% | 0.00% | 0.00% | 0.00% |
| **16.f.Muribaculaceae.g.Muribaculaceae** | 0.00% | 0.00% | 0.00% | 0.00% | 0.00% | 0.00% | 0.00% | 0.00% |
| **73.f.Muribaculaceae.g.Muribaculaceae** | 0.02% | 0.00% | 0.00% | 0.00% | 0.00% | 0.00% | 0.00% | 0.00% |
| **113.f.Muribaculaceae.g.Muribaculaceae** | 0.00% | 0.00% | 0.00% | 0.00% | 0.00% | 0.00% | 0.00% | 0.00% |
| **119.f.Muribaculaceae.g.Muribaculaceae** | 0.00% | 0.00% | 0.00% | 0.00% | 0.00% | 0.00% | 0.00% | 0.00% |
| **168.f.Muribaculaceae.g.Muribaculaceae** | 1.54% | 1.78% | 2.03% | 3.09% | 0.05% | 0.00% | 0.00% | 0.00% |
| **185.f.Muribaculaceae.g.Muribaculaceae** | 0.00% | 0.00% | 0.00% | 0.00% | 0.00% | 0.00% | 0.00% | 0.00% |
| **191.f.Muribaculaceae.g.Muribaculaceae** | 0.00% | 0.00% | 0.00% | 0.00% | 0.00% | 0.00% | 0.00% | 0.00% |
| **225.f.Muribaculaceae.g.Muribaculaceae** | 0.00% | 0.00% | 0.00% | 0.00% | 0.00% | 0.00% | 0.00% | 0.00% |
| **231.f.Muribaculaceae.g.Muribaculaceae** | 0.00% | 0.00% | 0.00% | 0.00% | 0.00% | 0.00% | 0.00% | 0.00% |
| **312.f.Muribaculaceae.g.Muribaculaceae** | 0.00% | 0.00% | 0.00% | 0.00% | 0.00% | 0.00% | 0.00% | 0.00% |
| **328.f.Muribaculaceae.g.Muribaculaceae** | 0.00% | 0.00% | 0.00% | 0.00% | 0.00% | 0.00% | 0.00% | 0.00% |
| **417.f.Muribaculaceae.g.Muribaculaceae** | 0.00% | 0.00% | 0.00% | 0.00% | 0.00% | 0.00% | 0.00% | 0.00% |
| **418.f.Muribaculaceae.g.Muribaculaceae** | 0.00% | 0.00% | 0.00% | 0.00% | 0.00% | 0.00% | 0.00% | 0.00% |
| **501.f.Muribaculaceae.g.Muribaculaceae** | 0.00% | 0.00% | 0.00% | 0.00% | 0.00% | 0.00% | 0.00% | 0.00% |
| **573.f.Muribaculaceae.g.Muribaculaceae** | 0.00% | 0.00% | 0.00% | 0.00% | 0.00% | 0.00% | 0.00% | 0.00% |
| **577.f.Muribaculaceae.g.Muribaculaceae** | 0.28% | 0.51% | 0.75% | 0.92% | 0.02% | 0.00% | 0.00% | 0.00% |
| **585.f.Muribaculaceae.g.Muribaculaceae** | 0.00% | 0.00% | 0.00% | 0.00% | 0.00% | 0.00% | 0.00% | 0.00% |
| **592.f.Muribaculaceae.g.Muribaculaceae** | 0.00% | 0.00% | 0.00% | 0.00% | 0.00% | 0.00% | 0.00% | 0.00% |
| **616.f.Muribaculaceae.g.Muribaculaceae** | 0.44% | 0.37% | 0.61% | 0.84% | 0.02% | 0.00% | 0.00% | 0.00% |
| **640.f.Muribaculaceae.g.Muribaculaceae** | 0.34% | 0.11% | 0.10% | 0.05% | 0.00% | 0.00% | 0.00% | 0.00% |
| **675.f.Muribaculaceae.g.Muribaculaceae** | 0.92% | 0.91% | 0.01% | 1.23% | 0.06% | 0.00% | 0.00% | 0.00% |
| **728.f.Muribaculaceae.g.Muribaculaceae** | 0.00% | 0.00% | 0.00% | 0.00% | 0.00% | 0.00% | 0.00% | 0.00% |
| **743.f.Muribaculaceae.g.Muribaculaceae** | 2.66% | 3.36% | 3.84% | 8.77% | 0.16% | 0.00% | 0.00% | 0.00% |
| **750.f.Muribaculaceae.g.Muribaculaceae** | 0.84% | 0.94% | 0.01% | 1.15% | 0.05% | 0.00% | 0.00% | 0.00% |
| **773.f.Muribaculaceae.g.Muribaculaceae** | 0.00% | 0.00% | 0.00% | 0.00% | 0.00% | 0.00% | 0.00% | 0.00% |
| **815.f.Muribaculaceae.g.Muribaculaceae** | 0.89% | 3.14% | 2.29% | 6.85% | 0.20% | 0.00% | 0.00% | 0.00% |
| **934.f.Muribaculaceae.g.Muribaculaceae** | 0.00% | 0.00% | 0.00% | 0.00% | 0.00% | 0.00% | 0.00% | 0.00% |
| **944.f.Muribaculaceae.g.Muribaculaceae** | 2.88% | 1.55% | 1.15% | 1.17% | 0.02% | 0.00% | 0.00% | 0.00% |
| **964.f.Muribaculaceae.g.Muribaculaceae** | 0.18% | 0.20% | 0.31% | 0.42% | 0.02% | 0.00% | 0.00% | 0.00% |
| **974.f.Muribaculaceae.g.Muribaculaceae** | 0.00% | 0.00% | 0.00% | 0.00% | 0.00% | 0.00% | 0.00% | 0.00% |

**Supplementary Figure S9. |** Relative abundance of *Muribaculaceae* in untreated and treated Hom mice with table detailing the relative abundance percentages. Reds indicate ASVs with highest sequence identity to *Duncaniella muris*. Blues indicate ASVs with secondary and tertiary sequence identities to *Duncaniella muris*. Percentages below Age in Weeks indicates relative abundance of *Muribaculaceae*. *n* = 8-12 per group.
