# Supplementary material for: Perinatal antibiotics in a db/db mouse model impact obesity and hyperglycemia in a microbiome-dependent manner
Source: Front Microbiomes. 2026 Jul 27;5:1750320. doi: 10.3389/frmbi.2026.1750320 (PMC13454812; doi:10.3389/frmbi.2026.1750320)
Supplement: Supplementary file 12 [file Supplementaryfile3.docx]

**Supplementary Figure S3. |** Caloric density of desiccated feces. *n* = 5 per group.
